# Valorization of Rapeseed Meal: Production, Fractionation, and Utilization Routes

**DOI:** 10.3390/foods15173082

**Published:** 2026-08-31

**Authors:** Xiaoxian Liu, Jin Xie, Christophe Blecker

**Affiliations:** 1 Unit of Food Science and Formulation, Gembloux Agro-Bio Tech, University of Liège, Avenue de la Faculté d’Agronomie 2B, B-5030 Gembloux, Belgium; xiaoxian.liu@doct.uliege.be (X.L.); christophe.blecker@uliege.be (C.B.); 2 Shenzhen International Graduate School, Tsinghua University, Shenzhen 518055, China

**Keywords:** rapeseed meal, biorefinery, fractionation, component recovery, integrated valorization, thermochemical conversion, protein, polysaccharide, phenolic compounds, glucosinolates

## Abstract

Rapeseed meal is a major by-product of rapeseed oil production and contains proteins, structural carbohydrates, and bioactive compounds with potential uses in feed, food ingredients, functional materials, and thermochemical products. However, broader valorization is constrained by variability in meal composition, interactions among components, fractionation efficiency, and scale-up requirements. This review examines how oil production routes affect meal composition and component accessibility, and summarizes physical fractionation, protein and polysaccharide recovery, and the separation, removal, or conversion of phenolic compounds, glucosinolates, and phytic acid. Applications of the resulting fractions in feed, fertilizer, foods, functional materials, bioactive products, and thermochemical conversion are evaluated. Existing evidence shows that pressing, solvent extraction, and thermal treatment alter residual oil content, protein solubility, protein–fiber associations, and the distribution of antinutritional compounds, thereby influencing subsequent separation. Fractionation conditions determine recovery, purity, structural state, and functionality and should be selected according to the intended product. Despite substantial progress, industrial implementation remains limited by feedstock variability, insufficient application-level validation, high water and chemical demand, and incomplete utilization of residual streams. Future work should define product-specific quality criteria, establish complete component mass balances, and develop continuous cascade fractionation processes with lower resource demand to support integrated and scalable rapeseed meal valorization.

## 1. Introduction

In recent decades, growing resource scarcity and waste generation have increased the need for more circular and efficient use of resources [1]. Circular economy and waste reduction strategies address this need by improving resource efficiency and reducing waste [2]. Within this resource-efficient approach, residues from agriculture and industry are increasingly regarded as renewable feedstocks for products with higher economic value.

With the growing interest in residue use, rapeseed meal has gained increasing attention as a renewable raw material. Rapeseed (*Brassica napus* L.) is the second-largest oil crop globally following soybean; global rapeseed meal production reached about 49.3 million metric tons in 2024/2025, making it one of the major oilseed meals worldwide [3]. Rapeseed meal is currently used mainly as animal feed, with some use as fertilizer [4]. However, rapeseed meal contains structural carbohydrates, proteins, phenolic compounds, phytic acid, glucosinolates (GLS), and other useful components that are not fully exploited by these conventional uses [5]. This limitation indicates that rapeseed meal still has further valorization potential.

**Figure 1:**
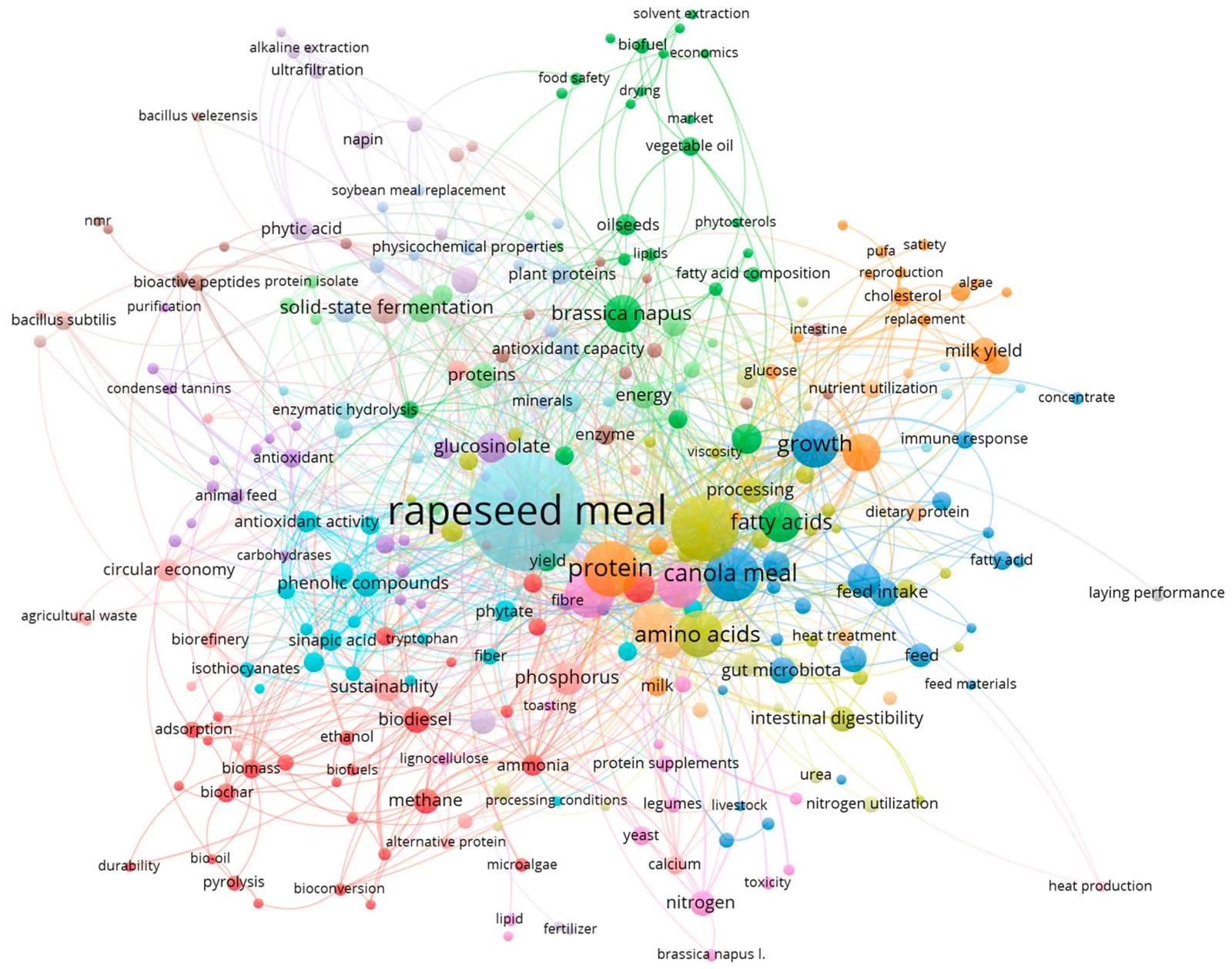
Network visualization (generated using VOSviewer) of research trends in rapeseed meal valorization, based on publications retrieved from the Web of Science Core Collection using the keyword “rapeseed meal”.

Rapeseed meal composition is influenced by both seed structure and processing conditions [6]. Processing steps such as pressing, solvent extraction, and thermal treatment determine the physical state of the meal and can affect the accessibility of its main components [7]. At the same time, rapeseed meal is a heterogeneous biomass because proteins, structural carbohydrates, phenolic compounds, phytic acid, GLS, and other components are not distributed in the same way within the seed tissues [8]. This heterogeneity makes fractionation important before targeted utilization. Fractionation strategies can redistribute or recover selected components, including proteins, polysaccharides, and phenolic compounds, while also supporting the management of phytic acid and GLS [5]. The resulting fractions can then be directed to different uses, including feed, fertilizer, food ingredients, functional materials, and thermochemical products [4,5]. These links among composition, fractionation, and utilization are also reflected in the VOSviewer_1.6.20 keyword co-occurrence network shown in Figure 1. Composition-related keywords, including protein, amino acids, fiber, GLS, phytic acid, and phenolic compounds, are closely associated with processing and fractionation approaches such as solid-state fermentation, enzymatic hydrolysis, alkaline extraction, and ultrafiltration. The network also covers major utilization themes, including feed applications, plant protein development, bioactive compound recovery, and biorefinery.

In recent years, several studies have summarized the valorization of rapeseed meal, mainly focusing on nutritional quality, bioactive compounds, and biorefinery strategies [5,9,10,11,12]. However, research on how rapeseed oil production affects meal composition and component accessibility, and how these effects are further related to subsequent fractionation and the intended applications of the resulting fractions, remains limited. Therefore, summarizing how processing and fractionation affect rapeseed meal composition and how the resulting fractions can be matched with suitable applications is important for improving the utilization of rapeseed meal.

The present review focuses on the links between upstream processing history, component accessibility, fractionation performance, and the suitability of the resulting fractions for specific downstream applications. Particular attention is given to the differences among recovery yield, purity and selectivity, product functionality, water, solvent, and energy demand, scalability, side-stream generation, and compatibility with subsequent processing. The available application evidence is further evaluated to distinguish routes that have been validated in food, feed, material, soil, or thermochemical systems from those that remain primarily at the laboratory stage, thereby providing guidance for the more efficient and sustainable use of rapeseed meal.

### Review Methodology

This review was conducted as a critical narrative review supported by a structured literature search. Relevant literature was searched using Web of Science, Scopus, ScienceDirect, and Google Scholar, with the final search conducted on 2 August 2026. The core terms “rapeseed meal”, “canola meal”, “rapeseed cake”, and “canola cake” were combined with topic-specific terms related to processing, fractionation, component recovery, functionality, and valorization. No fixed starting year was applied. Recent peer-reviewed studies were prioritized. Publications were selected according to their relevance to the scope and critical comparisons developed in this review.

## 2. Rapeseed Processing and Its Influence on Meal Characteristics

### 2.1. Conventional Rapeseed Oil Extraction Routes

**Figure 2:**
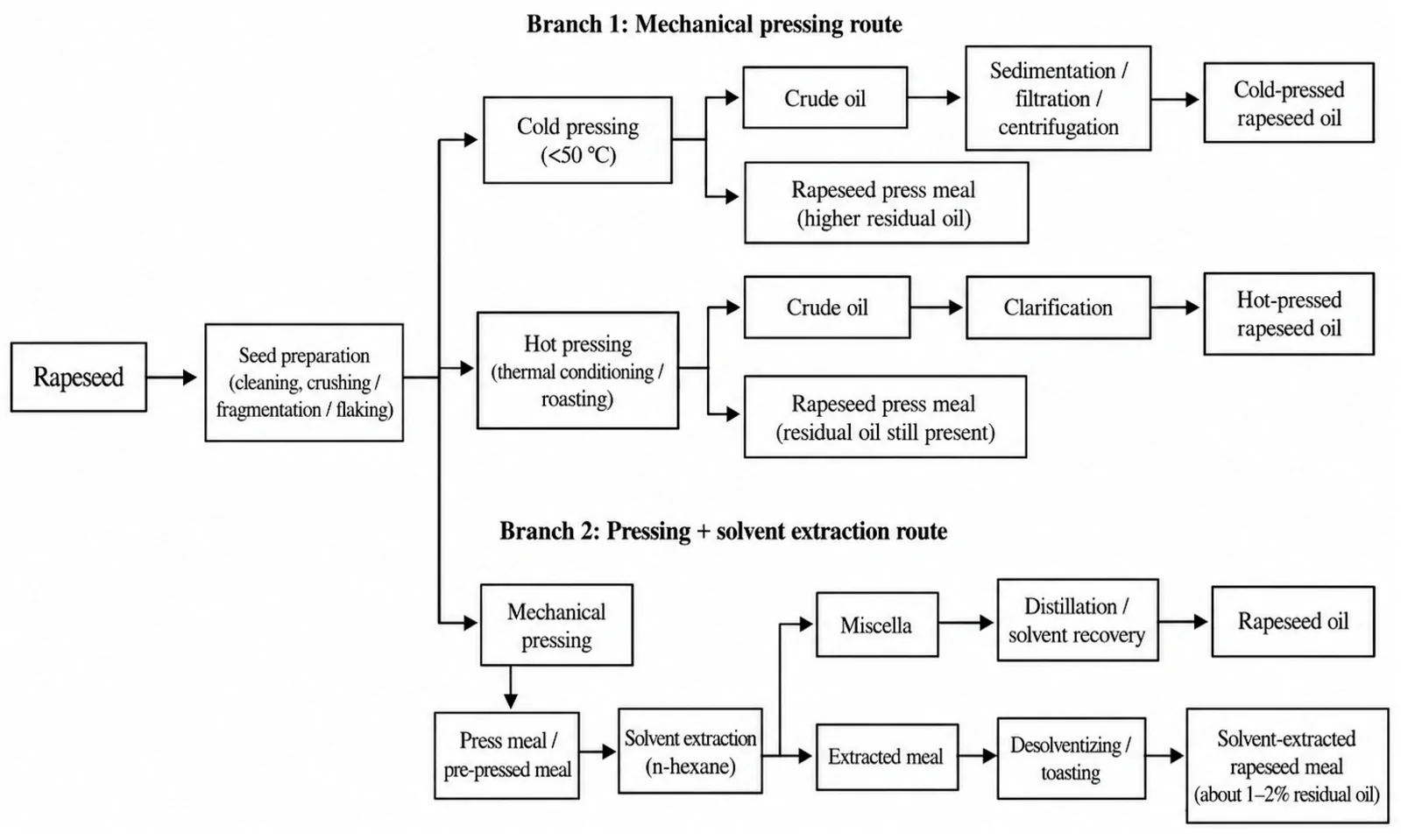
Main processing routes from rapeseed to rapeseed meal.

Conventional rapeseed oil extraction mainly follows two routes: mechanical pressing and solvent extraction [13]. Figure 2 summarizes the main processing routes from rapeseed to rapeseed meal. Cold pressing imposes relatively mild thermal exposure but generally leaves more residual oil, whereas hot pressing increases thermal exposure of the solid material. Solvent extraction further removes residual oil and is followed by desolventizing/toasting. The differences in processing conditions and thermal exposure can further influence the characteristics of the resulting meal.

### 2.2. Influence of Processing Conditions on Meal Composition and Component Accessibility

Different extraction routes lead to different compositions of rapeseed meal by changing residual oil content and the retention or removal of non-lipid components. Mechanical pressing generally leaves more residual oil in rapeseed press meal, whereas subsequent solvent extraction removes additional oil and increases the relative proportions of protein, fiber, ash, and other non-lipid components on a dry matter basis [6]. Mosenthin, Messerschmidt, Sauer, Carré, Quinsac, and Schöne [6] followed a single batch of rapeseed through pressing, solvent extraction, and desolventizing/toasting and compared the solid materials collected at different processing stages. The rapeseed press meal contained 210 g/kg dry matter of oil and 294 g/kg dry matter of crude protein, whereas the solvent-extracted meals contained only 18–23 g/kg dry matter of oil and 376–382 g/kg dry matter of crude protein. This shift mainly reflects the relative concentration of non-lipid components following oil removal, rather than an absolute increase in the amount of protein or fiber in the original material.

Solvent properties can also affect the retention or removal of non-lipid components. Proteins, GLS, phenolic compounds, and other soluble compounds differ in polarity, molecular size, and solubility in the extraction medium, so changes in solvent polarity and water content can alter their retention in the meal to different extents. Citeau, Regis, Carré, and Fine [14] compared hexane, ethanol, and isopropanol for rapeseed oil extraction and found that hydroalcoholic solvents increased the protein content of the resulting meal by approximately 13% compared with hexane extraction. Increasing the water content of the solvent also promoted GLS extraction, and isopropanol containing 84 wt.% isopropanol produced meals with GLS concentrations 49–73% lower than those obtained using the other alcohol systems. These results show that solvent polarity and water content can affect meal composition in two connected ways: they can increase the relative proportion of proteins that largely remain in the solid matrix, while also promoting the removal of more solvent-sensitive polar compounds such as GLS. Albe-Slabi et al. [15] further indicated that cold-pressed rapeseed meal, which does not undergo solvent extraction and desolventizing, can retain and concentrate GLS and endogenous myrosinase activity. This indicates that milder oil extraction may preserve more reactive polar compounds and related endogenous enzymes, which need to be considered when selecting subsequent utilization routes. Therefore, the effect of oil extraction on rapeseed meal composition depends on both the degree of oil removal and the selectivity of the solvent toward non-lipid components.

Thermal and mechanical conditions of processing can further affect the structural state, component associations, and accessibility of meal components. Cold-pressed rapeseed meal experiences relatively mild thermal treatment, whereas industrial pre-pressing, hot pressing, and solvent removal after extraction increase the thermal exposure of the solid material [7,16]. This effect is particularly evident for proteins, because heating and mechanical compaction can alter protein conformation and solubility while strengthening the association between proteins and the fiber matrix. Fetzer, Herfellner, Stäbler, Menner, and Eisner [7] similarly found that the more strongly heated, pre-pressed meal had a lower protein extraction yield. Under mild extraction conditions, the maximum protein extraction yields from cold-pressed and pre-pressed meals were 52.3% and 36.7%, respectively. Östbring, Malmqvist, Nilsson, Rosenlind, and Rayner [16] later applied the same protein recovery procedure to cold-pressed, hot-pressed, and solvent-extracted rapeseed meal. Without additional heating during protein recovery, the corresponding protein recovery yields were 45%, 26%, and 5%, respectively, further demonstrating that the processing conditions during oil production strongly influence subsequent protein recovery. More recently, this temperature-dependent effect was demonstrated in rapeseed meals pressed at 75 and 150 °C, where the higher temperature promoted the association of part of the protein with the fiber matrix, reduced protein solubility and α-helix content, and consequently hindered subsequent protein extraction [17]. Matrix structure also affects this process. Baker et al. [18] showed that upstream mechanical treatment, such as wet milling, improved protein release from cold- and hot-pressed rapeseed meal, indicating that protein recovery is not governed only by protein properties, but is also limited by particle disruption and matrix accessibility. Alpiger, Politiek, Corredig, Schutyser, and van der Goot [19] further found that rapeseed press meal produced at different pressing temperatures had different residual oil levels and structural characteristics, which influenced protein enrichment from the solid matrix. These studies indicate that processing conditions can reorganize the internal matrix of rapeseed meal by altering protein conformation and solubility, protein–fiber associations, particle structure, and residual oil distribution, thereby affecting component digestibility, accessibility, and subsequent recovery.

Desolventizing and toasting after solvent extraction further affect the composition and quality of the final rapeseed meal. Heat and steam are applied to remove residual extraction solvent, while temperature, steam conditions, and residence time in the desolventizer/toaster are the main controlling factors. Mosenthin, Messerschmidt, Sauer, Carré, Quinsac, and Schöne [6] showed that longer heat treatment increased the measured contents of neutral detergent fiber and neutral detergent fiber-bound crude protein, while decreasing lysine, reactive lysine, cysteine, and protein solubility. These changes are associated with stronger interactions between protein and fiber and with Maillard reactions [20]. The free amino group of lysine is particularly susceptible to reactions with reducing sugars, which produce bound forms with lower biological availability. In addition to these effects on protein quality, increasing the intensity of desolventizing and toasting promotes the degradation of heat-sensitive GLS. Mosenthin, Messerschmidt, Sauer, Carré, Quinsac, and Schöne [6] found that extending the residence time under wet toasting conditions from 48 to 93 min increased total GLS degradation from 54% to 83%, while subsequent dry heat treatment increased the degradation to 89%. The intensity of desolventizing and toasting therefore determines the final state of several meal components, including the degree of GLS degradation, protein aggregation, amino acid integrity, protein solubility, and nutritional availability.

Accordingly, the processing history of rapeseed meal should be considered when selecting subsequent fractionation strategies, as differences in residual oil content, protein accessibility, and thermal exposure can substantially influence separation performance.

## 3. Fractionation Strategies for Rapeseed Meal Valorization

### 3.1. Physical Fractionation by Dehulling Before and After Oil Extraction

#### 3.1.1. Front-End Dehulling

Rapeseed hull and cotyledon tissues have different structural and physical characteristics. The hull accounts for approximately 18.2% of rapeseed mass but contains about 73% of the neutral detergent fiber, 80% of the acid detergent fiber, and 95% of the lignin present in the whole seed, whereas protein and oil are mainly located in the cotyledon tissue [21]. Differences in mechanical strength, deformation behavior, particle size, shape, and aerodynamic properties provide the basis for mechanical hull disruption and physical separation of the two tissues [21,22]. According to the stage at which dehulling is introduced into the oil extraction process, it can be classified as front-end or tail-end dehulling [23]. Front-end dehulling is applied to intact rapeseeds and separates the hull and cotyledon before pressing or solvent extraction [24]. Tail-end dehulling is applied to rapeseed meal and separates hull and cotyledon particles through milling and particle classification [23].

Front-end dehulling generally consists of conditioning, mechanical hull disruption, and hull–cotyledon separation. Conditioning refers to the adjustment of seed moisture and temperature before mechanical treatment. This operation changes the brittleness, elasticity, and interfacial association of the hull and cotyledon, allowing subsequent mechanical loading to cause hull cracking more readily. Ikebudu et al. [25] increased the moisture content of rapeseeds from approximately 7.5% to 15%, allowed them to equilibrate for 10 min, and then dried them at 70–75 °C for 5 min. This treatment produced a dehulling index of 0.88, compared with −0.47 for the untreated control. When the moistening period was extended to 15 min and seed moisture increased to 18.5%, the index decreased to 0.83 because the water absorption increased the elasticity and toughness of the hull, making brittle fracture more difficult during abrasion. Martinez-Soberanes et al. [26] further compared conventional oven, microwave, and fluidized-bed drying after seed moistening. Fluidized-bed drying produced more pronounced shrinkage of the internal kernel and a clearer separation between the hull and kernel than oven or microwave drying, thereby facilitating subsequent dehulling. These results indicate that conditioning performance depends not only on seed moisture adjustment, but also on the differential structural changes induced by the drying method.

Mechanical disruption must sufficiently crack the hull while limiting the crushing of the oil-rich cotyledon and the generation of fines. Ikebudu, Sokhansanj, Tyler, Milne, and Thakor [25] treated approximately 20 g of seeds for 60 s using a tangential abrasive disc with a grit grade of 80. The abraded material was first separated by air aspiration, after which sieving was used to further separate hull particles from fines. After heating at 120 °C, 20.01 g of seeds produced 16.09 g of cotyledons, 2.80 g of hulls, 0.81 g of undehulled seeds, and 0.47 g of fines, corresponding to approximately 80.4%, 14.0%, 4.0%, and 2.3% of the feed mass, respectively. At 60 °C, the hull fraction was 2.49 g, the fine fraction was 0.97 g, and the undehulled seed fraction was 1.31 g. Increasing the treatment temperature from 60 to 120 °C increased hull recovery by approximately 13%, reduced fine generation by approximately 50%, and decreased the proportion of undehulled seeds. The direction of mechanical loading also affects hull rupture and cotyledon damage. Martinez-Soberanes, Purdy, Reaney, and Zhang [21] conducted compression and shear tests on individual seeds over a moisture range of 5.0–25.0%. At the same moisture content, the ultimate shear strength was approximately 1/4 of the ultimate compressive strength, indicating that shear loading could rupture the hull at a lower applied stress. Morphological observations showed that, at 5.8% moisture, the cotyledon largely retained its original shape after shear-induced hull rupture, whereas compression and higher moisture contents caused more pronounced flattening and plastic deformation of the cotyledon.

After mechanical disruption, the released hull and cotyledon materials are commonly separated by air and sieving. The main difficulty is that thin outer cotyledon fragments can resemble hull pieces in size and shape and may therefore enter the hull fraction during air separation. Carré and Loison [27] therefore introduced an additional roller purification step after aspiration, in which cotyledon particles were flattened and retained on the metal roll surface, while most hull particles passed through the 0.1 mm gap. Under the selected feed rate and roll speed, the hull and cotyledon fractions reached purities of 96% and 94%, respectively. Yin et al. [28] instead improved the release of the hull before separation by moistening the rapeseeds and then rapidly drying them in a fluidized bed. The drying consisted of heating at 50 °C for 10 min with an air velocity of 6.5 m/s, followed by cooling at room temperature for 5 min. The best group was obtained at a final rapeseed moisture content of 11%, where the dehulling efficacy reached 98%.

Front-end dehulling can reduce the proportion of hulls before oil extraction and improve the protein and fiber composition of the resulting meal. However, removing the hull fraction before oil extraction causes some oil loss and may affect subsequent pressing. In comparison, tail-end dehulling is applied to rapeseed meal after oil extraction and therefore does not directly affect the preceding oil recovery process.

#### 3.1.2. Tail-End Dehulling

Tail-end dehulling is applied to rapeseed meal after oil extraction. Rapeseed meal commonly contains hull fragments, cotyledon particles, and composite particles in which the two tissues remain incompletely separated [24]. Tail-end dehulling therefore usually begins with milling to promote the dissociation of hull and cotyledon [24]. Cotyledon is relatively brittle and readily forms fine protein-rich particles after milling, whereas hull, which contains higher levels of fiber and lignin, can remain as relatively large particles under suitable milling conditions. The particle size distributions of the two tissues and the extent of their overlap determine the subsequent separation method. Sieving can be applied when the particle size difference is clear, whereas air classification, density separation, or electrostatic separation is required when the particle sizes overlap substantially [29].

Milling–sieving uses the different breakage behaviors of hull and cotyledon. Cotyledon particles enter the protein-rich fine fraction, whereas larger hull fragments remain in the coarse fraction. Mejicanos, Rogiewicz, Nyachoti, and Slominski [23] used 250–600 μm sieves to process conventional black-seeded *Brassica napus* meal, yellow-seeded *B. napus* meal, and *B. juncea* meal. The fine fractions from all three materials showed increased crude protein and decreased total dietary fiber. In the finest fractions, crude protein increased from 368, 410, and 423 g/kg to 420, 436, and 479 g/kg, respectively, while total dietary fiber decreased from 300, 270, and 255 g/kg to 214, 216, and 153 g/kg, respectively. *B. juncea* meal showed the greatest reduction in fiber and the highest final protein content, indicating that the separation outcome was also affected by tissue structure and the particle size distribution produced during milling. The milling method further determines whether hull and cotyledon develop particle size differences that can be recognized by the sieve. Hansen, Skrede, Mydland, and Øverland [8] found that the 0–150 μm fraction obtained by ball milling–sieving represented 42.3% of the original material, with crude protein increasing from 336 to 394 g/kg. Repeated impact during ball milling promoted the release of cotyledon while retaining some larger hull particles that could be intercepted by the sieve. Bárta et al. [30] used a single 250 μm sieve to process rapeseed meal. Material loss during sieving was low, and the mass yields of both the coarse and fine fractions were close to 50%, providing sufficient quantities of both fractions. A significant difference in protein content was also observed between the two fractions, showing that a 250 μm single sieving step could achieve effective compositional separation.

Milling–air classification separates particles according to their movement in an air stream and centrifugal field. Particle allocation could be affected by particle size, density, shape, and aggregation state [24,31]. To reduce particle adhesion and aggregation caused by residual oil, Partanen et al. [32] first defatted rapeseed press meal using supercritical CO_2_ and then applied milling and air classification. The protein content increased from 35.9% to 45.8%. Defatting improved the dispersion of fine particles and allowed the aerodynamic differences between particles from different tissues to be used more effectively. Industrial air classification also requires the rotor speed and airflow conditions to be matched to the particle size distribution of the feed. Rempel et al. [33] found that rotor speed controlled the range of particles entering the fine fraction by changing the centrifugal force and classification cut point. Secondary air improved feed dispersion and reduced particle aggregation, and both factors affected the protein content of the fine fraction. The main airflow further regulated particle movement and residence within the classifier, thereby changing the mass distribution between the fine and coarse fractions. Wockenfuss et al. [34] compared air classification and electrostatic separation and found that both methods produced a protein-rich fraction from defatted rapeseed press meal. Air classification separates particles according to their aerodynamic behavior and can recover a relatively large amount of fine fraction. However, some fine hull particles also enter the target fraction, resulting in lower protein content than that obtained by electrostatic separation. Air classification is suitable for continuous processing and can provide relatively high yield of the target fraction, although its performance is sensitive to powder dispersion and operating parameters.

Milling–electrostatic separation uses differences in the electrical charges generated by protein particles and lignocellulosic particles during contact and friction. Therefore, both tissue dissociation and exposure of fresh particle surfaces need to be achieved by milling [34]. Basset et al. [35] combined ultrafine milling with triboelectrostatic separation and increased the protein content of defatted rapeseed meal from approximately 37% to 47%, with a mass yield of about 39% for the fine fraction. Particle size is not the only factor determining electrostatic separation performance, as the surface characteristics created by milling are also important. Kdidi, Vaca-Medina, Peydecastaing, Oukarroum, Fayoud, and Barakat [29] compared impact milling, ball milling, and jet milling and found that impact milling combined with electrostatic separation produced better separation of protein and lignocellulosic particles. This result indicates that the milling method affects the exposure of surface components and the triboelectric behavior of the particles. Laguna et al. [36] compared electrostatic and turbo separation for ultrafine-milled rapeseed meal. Ultrafine milling reduced both hull and cotyledon to fine particles, thereby decreasing the original differences in particle size and density between the two tissues. Turbo separation therefore produced relatively limited compositional changes. Electrostatic separation could still use differences in surface composition and charging behavior between protein particles and lignocellulosic particles, resulting in greater enrichment of both protein and phenolic compounds. Pressing conditions can also affect electrostatic separation through differences in residual oil and particle structure. Alpiger, Politiek, Corredig, Schutyser, and van der Goot [19] compared rapeseed meal produced at pressing temperatures of 60–130 °C and found relative protein enrichment of 17–30% after milling and electrostatic separation. The meal produced at 80 °C had lower residual oil content and achieved relatively high protein recovery. Electrostatic separation has high selectivity for particles with similar sizes but different surface compositions and is therefore suitable for further protein enrichment when the material has been sufficiently milled and remains well dispersed.

Density separation and combined processes can be used to treat fractions that still contain substantial amounts of composite or incorrectly allocated particles after the first separation step. Wockenfuss, Lammers, Heinz, Sozer, and Silventoinen-Veijalainen [34] reprocessed the coarse fraction produced in the first separation step and increased its protein content from 32% to 37–38%, with a maximum protein separation efficiency of approximately 70%. In contrast, reprocessing the already fine fraction produced only minor compositional changes. The main value of combined classification therefore lies in recovering protein particles that had already been released but were incorrectly allocated to the coarse fraction during the first separation step. Increasing the number of classification steps is unlikely to improve the recovery of protein that remains embedded in hull–cotyledon composite particles, and further dissociation is required.

Overall, tail-end dehulling first requires sufficient dissociation of the hull and cotyledon. After milling, the separation method can be selected according to the dominant particle difference: size for sieving, aerodynamic behavior for air classification, and surface charge for electrostatic separation. Reprocessing is useful mainly for released particles that were misclassified in the first separation step.

### 3.2. Polysaccharide Recovery

Fractionation of rapeseed meal polysaccharides requires disruption of the interactions among cell wall polysaccharides and between polysaccharides and other matrix components, particularly proteins [37]. Different polysaccharides can then be selectively transferred into the liquid phase and subsequently separated and purified according to differences in solubility, charge, or molecular size [38]. Rapeseed meal cell walls contain pectic polysaccharides, arabinan, arabinogalactan, xyloglucan, xylan, cellulose, and other polysaccharides with different solubilities and binding states [9]. The extraction agent determines the types of polysaccharides that can be solubilized, while pH, temperature, and extraction time further affect their release, molecular weight, and structural integrity [39].

Hot water extraction is a relatively mild method and is mainly used to recover water-soluble polysaccharides or polysaccharides weakly associated with the cell wall. Pustjens et al. [40] extracted rapeseed meal with hot water at 70 °C. Approximately 26% of the dry matter and 27% of the total sugars were transferred into the extract, while substantial amounts of xyloglucan, pectic polysaccharides, and cellulose remained in the residue. Hot water extraction helps preserve polysaccharide structure but has limited ability to release polysaccharides that are tightly associated with the cell wall.

Chemical extraction includes acid and alkaline extraction. Acid extraction is mainly used to promote the release of pectic polysaccharides. Jeong et al. [41] compared defatting, acid treatment, and enzyme treatment for pectin extraction from rapeseed meal, with 1% HCl used for the acid treatment. Defatting and enzyme treatment increased both pectin yield and galacturonic acid content, whereas severe acid treatment caused galacturonic acid degradation and reduced the pectin yield. Increasing acidity or temperature can enhance cell wall matrix disruption, but it can also accelerate glycosidic bond hydrolysis, resulting in galacturonic acid loss and a decrease in polysaccharide molecular weight [39]. Acid extraction conditions should therefore be selected by considering polysaccharide recovery, galacturonic acid retention, and molecular weight distribution together. Alkaline extraction promotes cell wall swelling and weakens the interactions of polysaccharides with proteins and other cell wall components. This method can therefore release tightly bound polysaccharides that remain in the solid residue after hot water extraction. Zhu and Wu [42] first treated defatted rapeseed meal with 80% ethanol to remove part of the phenolic compounds and low-molecular-weight sugars. Sequential hot water and alkaline extraction, followed by purification with D3520 macroporous resin, ethanol precipitation, and DEAE-52 cellulose chromatography, produced water-soluble polysaccharide, WPS-1, and an alkali-soluble polysaccharide, APS-2. The average molecular weights of WPS-1 and APS-2 were 7.20 × 10^5^ and 1.61 × 10^5^ Da, respectively. Arabinose, galactose, and glucose were the major monosaccharides in both products, although the two polysaccharides differed in molecular weight and the extent of protein association. Crude polysaccharides obtained by dilute alkaline extraction may still contain components with different charges and chemical compositions. Sun et al. [43] used 0.3% NaOH to extract crude polysaccharides from rapeseed meal and subsequently separated two fractions, PRM1 and PRM2, using DEAE cellulose DE-52 chromatography. The two components differed in their sugar, protein, and uronic acid contents and showed different radical scavenging, reducing, and lipid peroxidation inhibitory activities. Acid and alkaline extraction can also be combined to separate cell wall polysaccharides with different solubilities and binding states. Ghosh et al. [44] treated defatted rapeseed meal sequentially with acid and different alkaline solutions. The combined amount of acid-soluble and dilute alkali-soluble polysaccharides reached 270 mg/g of raw material and mainly consisted of arabinan, rhamnogalacturonan I, and arabinogalactan protein. Further extraction with 4 M KOH produced a xyloglucan dominated by XXXG-type structural units.

Enzyme-assisted extraction selectively degrades proteins and cell wall structures that restrict polysaccharide release and can promote solubilization under relatively mild conditions. Jeong et al. [45] treated rapeseed meal with a combination of Celluclast and Alcalase. The pectin yield of 6.85% was obtained at an enzyme-to-substrate ratio of 1:50, hydrolysis time of 270 min, and Celluclast-to-Alcalase ratio of 1:4. Protease treatment weakened protein–polysaccharide associations, while cellulase increased the accessibility of the cell wall matrix. Tian et al. [46] also observed changes in the distribution of carbohydrates, proteins, and other components between the aqueous phase and the solid residue after mixed enzyme treatment of rapeseed press meal. Banjanac et al. [47] further developed a two-step enzyme-assisted process for recovering pectic polysaccharides from rapeseed meal. Alcalase treatment for 2 h was followed by treatments with cellulase and hemicellulase preparations for 24 h, after which the remaining calcium-bound pectin was extracted with ammonium oxalate. Two fractions enriched in pectic polysaccharides were obtained, with yields of 6.8% and 2.7%, respectively. The sequential combination of protein hydrolysis, cell wall degradation, and calcium-bound pectin release provides a route for improving the selective recovery of pectic polysaccharides.

Crude polysaccharide extracts obtained from rapeseed meal generally contain proteins, pigments, phenolic compounds, minerals, and low-molecular-weight sugars. Additional concentration and purification are therefore required through precipitation, adsorption, ion exchange, or membrane separation [48]. Ethanol precipitation is simple and suitable for preliminary recovery of crude polysaccharides, but provides limited selectivity between polysaccharides with similar compositions and molecular weights. Duan et al. [49] compared HP-20, D3520, XAD-16, and AB-8 macroporous resins for deproteinization of rapeseed meal polysaccharides. HP-20 provided a suitable balance between protein removal and polysaccharide retention. Under dynamic adsorption and desorption conditions, the protein removal rate reached 91%, while the polysaccharide recovery was 62%. Ion exchange chromatography can separate neutral and acidic polysaccharides according to charge, while gel filtration can further narrow the molecular weight distribution. Ultrafiltration is suitable for concentrating crude polysaccharides and separating them according to molecular size. Sun et al. [50] compared ultrafiltration membranes with molecular weight cutoffs of 3, 8, and 12 kDa. The 3 kDa membrane produced the highest polysaccharide recovery while maintaining relatively high purity. Transmembrane pressure, extract pH, and temperature significantly affected membrane flux, whereas ionic content had a smaller effect. Ultrafiltration can also remove part of the monosaccharides, oligosaccharides, pigments, and minerals, although membrane fouling and overlapping molecular weight distributions can reduce separation efficiency.

The evaluation of polysaccharide extraction processes generally considers extraction yield, selectivity, structural preservation, solvent and energy consumption, and potential for scale-up. The resulting products should also be evaluated in terms of purity, monosaccharide composition, uronic acid content, molecular weight, molecular conformation, and functional properties. Hot water extraction is suitable for relatively soluble polysaccharides, acid extraction is mainly applied to pectic polysaccharides, and alkaline extraction can release tightly bound pectic and hemicellulosic polysaccharides [51,52,53]. Enzyme-assisted extraction provides advantages in selectivity and structural preservation. Subsequent ethanol precipitation, resin adsorption, ion exchange, and membrane separation further determine polysaccharide purity, composition, and molecular weight distribution. Polysaccharides are the major components of rapeseed meal. Current studies have mainly addressed the composition and structural characteristics of cell wall polysaccharides [40,44], the extraction and purification of polysaccharides [41,42,45,47,49,50,54], and the evaluation of antioxidant activity and prebiotic potential [42,43,55]. Further research could provide a more systematic understanding of how extraction conditions affect polysaccharide recovery, molecular structure, and product purity, while also considering chemical consumption, water use, and process scalability.

### 3.3. Protein Enrichment and Recovery

#### 3.3.1. Protein Extraction

Protein extraction from rapeseed meal is generally carried out in an aqueous medium to solubilize proteins from the solid matrix into the liquid phase [56]. Extraction performance is influenced by the extraction medium, operating conditions, and raw material characteristics [57].

Alkaline conditions increase the net negative charge and electrostatic repulsion between protein molecules, thereby promoting protein solubilization in the aqueous phase [58]. Ghodsvali et al. [59] extracted proteins from rapeseed meal at pH 9.5–12.0 and established a basic process combining alkaline extraction with subsequent precipitation or membrane separation. Gerzhova et al. [60] further compared the effects of pH, meal concentration, and NaCl addition and identified pH as the main factor affecting protein extraction. The highest extraction yield of 58.12% was obtained at pH 12 with a meal concentration of 15% and without added salt. Salt addition increased cruciferin solubility but reduced napin extraction. Fetzer, Herfellner, Stäbler, Menner, and Eisner [7] extended the comparison to the solid-to-liquid ratio, temperature, extraction time, and number of extraction cycles. Extraction at the native pH of 5.7–5.8 approached that obtained at pH 7–9, while NaCl concentration was a key parameter under mild extraction conditions. The maximum extraction yields from cold-pressed and pre-pressed rapeseed meals were 52.3% and 36.7%, respectively. Östbring et al. [61] used a solid-to-liquid ratio of 1:10 (*w*/*w*), extracted at pH 10.5 and 4 °C for 3 h, and precipitated the proteins at pH 5.0. When five rapeseed cultivars and one blended sample were processed under these conditions, protein recovery still varied from 26% to 41%, indicating that raw material differences also affect initial protein extractability. Zhang et al. [62] subsequently investigated a wider pH range and found that extraction at pH 9 followed by precipitation at pH 4.5 gave a product yield of 65.08%, while maintaining protein structure and limiting color darkening and the co-extraction of antinutritional compounds.

Although increasing pH generally promotes protein solubilization, strong alkaline conditions can also increase conformational changes and the co-extraction of non-protein compounds [62]. To reduce the adverse effects of strong alkaline extraction on product quality, Zhang, Fang, Feng, Chen, Qiu, Sun, Wu, and He [62] applied weakly acidic salt extraction at pH 6.5 with 150 mmol/L MgCl_2_. Protein extraction exceeded 40% for all salt-based routes, and the resulting products showed better color, odor, solubility, emulsifying properties, and digestibility than conventionally alkaline-extracted proteins. Li et al. [63] further explained these differences based on protein composition. Salt-extracted proteins contained lower levels of GLS and phytate but were enriched in napin and other protease inhibitor proteins, whereas the alkaline-extracted protein showed an in vitro digestibility of 93%. Salt extraction therefore reduces exposure to strong alkaline conditions but also selectively alters the protein composition.

Enzyme-assisted extraction promotes protein release by disrupting cell wall structures or partially hydrolyzing proteins [64]. Rommi et al. [65] treated rapeseed press meal with pectinase, xylanase, and cellulase. Pectinase increased protein extraction by 1.7-fold compared with the untreated control, and up to 74% of the total protein was extracted from partially dehulled material. To reduce the high water requirement of mild aqueous extraction, Rommi et al. [66] subsequently applied pectinase treatment at high solid contents and obtained protein recovery of 40–41% at pH 6, which was comparable to non-enzymatic extraction at pH 10. Rodrigues et al. [67] then introduced phytase pretreatment, increasing protein extraction to 72.1% while reducing the phytate content of the resulting concentrate to approximately 1 g/kg. In contrast to cell wall-degrading enzymes and phytase, Fetzer, Herfellner, Stäbler, Menner, and Eisner [7] used protease treatment to increase the single-step extraction yield to approximately 60%, while three consecutive extraction cycles reached 78–81%, although the degree of protein hydrolysis also increased. Du, Sang, Yu, Zheng, Wang, Wang, and Chen [17] combined multi-enzyme hydrolysis with alkaline aqueous extraction, which further improved protein extraction, product yield, and solubility while reducing molecular weight and GLS content. Ghosh et al. [68] incorporated Alcalase treatment into reactive extrusion followed by a 5 min extraction at pH 9. Protein recovery increased by 48% and 42% compared with non-enzymatic alkaline extraction and conventional enzyme-assisted alkaline extraction, respectively, although the product contained approximately 49% protein and consisted mainly of partially hydrolyzed proteins. Enzyme-assisted extraction has therefore expanded from cell wall degradation to phytate removal, limited protein hydrolysis, and integration with mechanical processing, while increased extraction can be accompanied by changes in protein composition and molecular weight distribution. Enzyme-assisted extraction can enhance protein release through cell wall degradation, phytate hydrolysis, or limited proteolysis, and can also be combined with mechanical processing to intensify extraction.

Ultrasound and other physical intensification techniques promote protein release by improving particle disruption, mass transfer, and solvent penetration [69]. Dong et al. [70] combined ultrasound-assisted extraction with ultrafiltration and isoelectric precipitation. Ultrasound increased protein extraction efficiency, while ultrafiltration produced proteins with favorable functional properties. In contrast, Zhang, Fang, Feng, Chen, Qiu, Sun, Wu, and He [62] found that weakly acidic salt extraction combined with ultrafiltration without ultrasound produced better overall product quality than the ultrasound-assisted route. This result suggests that ultrasound mainly enhances protein release and mass transfer rather than final product quality. Dong et al. [71] further compared different combinations of enzyme treatment, ultrasound, and membrane separation. Both enzyme and ultrasound treatments increased protein purity, and membrane filtration further removed small molecular compounds and antinutritional factors, but enzyme pretreatment alone provided a better balance among product quality, cost, and process complexity. Beyond ultrasound-assisted extraction, pulsed electric field treatment increased protein extraction only from 29.27% to 31.81%, but altered protein secondary structure and solubility [72]. In comparison, pressurized liquid extraction reduced the processing time from 120 min to 6 min and achieved a liquid-phase protein recovery of 43.8%, although the optimum condition required a temperature of 150 °C [73]. Dhakal and Acharya [74] applied alkaline subcritical extraction at 160 °C and obtained relatively high protein recovery, but the amino acid composition was less favorable than that of the enzyme-extracted protein. These results indicate that shorter extraction times and higher recovery may be accompanied by quality changes caused by high-temperature treatment.

Deep eutectic solvents have recently been applied to extract rapeseed proteins under relatively mild or selective conditions [75]. Grudniewska et al. [76] used glycerol–choline chloride deep eutectic solvent to treat rapeseed meal and obtained a cruciferin-enriched precipitate containing approximately 40–50% protein, although the protein yield was around 20% and the extraction required relatively high temperature. Karimi et al. [77] further compared four natural deep eutectic solvents with alkaline extraction at pH 9 and pH 12. Protein extraction with the deep eutectic solvents ranged from 44% to 52.89%, exceeding the 39.14% obtained at pH 9 but remaining below the 68.34% obtained at pH 12. The resulting products showed a lighter color and smaller changes in protein structure. Đermanović et al. [78] also found that alkaline extraction at pH 12 gave higher protein extraction efficiency, whereas the choline chloride–urea system produced an isolate with protein purity of 95.8% despite its lower extraction yield. Karimi et al. [79] subsequently adjusted the solvent water content and introduced ultrasound, increasing the maximum extraction yields of two natural deep eutectic solvent systems from 50.28% and 45.48% to 60.68% and 58.11%, respectively, while reducing the co-extraction of phenolic compounds and phytate. These findings show that the performance of deep eutectic solvents is strongly influenced by solvent composition and water content, while ultrasound can further promote protein release. Compared with strong alkaline extraction, the main advantages of these solvents are extraction selectivity, product purity, and preservation of protein structure.

Overall, achieving high protein recovery, purity, structural integrity, and resource efficiency within the same extraction process remains challenging. Process selection therefore depends on the intended protein product and its compatibility with subsequent recovery and purification steps.

#### 3.3.2. Protein Recovery and Purification

After proteins are solubilized from the rapeseed meal matrix, the resulting extract generally contains residual oil, polysaccharides, minerals, phenolic compounds, and other low-molecular-weight components [80]. Further recovery and purification are therefore required to obtain protein-rich products. Isoelectric precipitation reduces protein solubility by adjusting the extract to a selected pH, whereas ultrafiltration and diafiltration retain proteins mainly according to molecular size while allowing part of the salts, sugars, and other small compounds to pass through the membrane [81]. Because cruciferin and napin differ in molecular size, isoelectric point, and solubility under acidic conditions, the selected recovery method also affects their relative proportions in the final product [11,82]. Isoelectric precipitation preferentially recovers cruciferin, whereas a substantial proportion of napin may remain in the acidic supernatant [83]. Membrane processing can recover these soluble proteins or provide an initial separation of the major storage protein fractions [84].

Isoelectric precipitation is the most commonly used method for recovering proteins from clarified rapeseed extracts; the extract is generally adjusted to pH 3.5–5.5, after which aggregated proteins are separated by centrifugation [85]. Östbring et al. [86] adjusted an alkaline extract from cold-pressed rapeseed meal to pH 6.5 and recovered approximately 38% of the soluble protein in the precipitate, corresponding to an overall protein recovery of 19%. Ahlström et al. [87] compared precipitation over a pH range of 3.0–6.5. Protein recovery reached approximately 33% at pH 3.5–4.0, whereas proteins precipitated at pH 5.0–6.0 produced more stable emulsions. Thus, the precipitation condition giving the highest recovery does not necessarily provide the most suitable functional properties. The practical limitation of incomplete protein precipitation was further demonstrated by Ahlström et al. [88] at pH 3.5; approximately 52% of the protein in the concentrated liquid phase entered the precipitate, while a further 18% remained in the low-protein supernatant. Most of the additional protein obtained by solids recirculation was recovered during the first two cycles, while further recirculation produced only limited improvement. Isoelectric precipitation therefore generates both a protein precipitate and a protein-containing acidic supernatant, and discarding the latter can substantially reduce overall recovery. Single precipitation pH mainly recovers proteins with similar solubility behavior. Sequential isoelectric precipitation has therefore been investigated to recover additional protein fractions and regulate product composition [89]. Kalaydzhiev et al. [90] compared stepwise precipitation from pH 10.5 to 2.5 with the reverse sequence from pH 2.5 to 8.5. The two routes produced isolates that differed in protein content, phenolic content, molecular-weight distribution, and solubility. Georgiev et al. [91] further found that these products differed in water- and oil-binding capacities, emulsifying and foaming properties, and antioxidant activity. Sequential precipitation can recover proteins that remain soluble at a single selected pH and can generate fractions with different compositions and functionalities. However, additional pH adjustments, centrifugation steps, and neutralization requirements increase chemical consumption and process complexity. Single precipitation is therefore more suitable for producing mixed protein ingredients, whereas sequential precipitation is more relevant when controlled fraction composition.

The retention of napin and other acid-soluble proteins in the precipitation supernatant provides a basis for combining precipitation with membrane processing. Tzeng et al. [92] established an early integrated process involving alkaline extraction, isoelectric precipitation, ultrafiltration, and diafiltration. Acid-insoluble proteins were first recovered by precipitation, while the remaining soluble proteins were concentrated from the supernatant by membrane filtration. This two-stream approach reduced protein losses in the acidic liquid fraction. Recently, membrane processing has also been used without initial precipitation. Jia et al. [93] prepared mildly refined rapeseed protein products by aqueous extraction and ultrafiltration. The resulting proteins retained a relatively native structure and showed approximately 78% solubility, although phenolic compounds were also retained to some extent. Cháirez-Jiménez et al. [94] compared several extraction and purification combinations and reported protein recovery of 34.88% for alkaline extraction followed by ultrafiltration. When ethanol pretreatment was introduced before alkaline extraction and ultrafiltration, the final protein content increased to 89.71%, whereas the precipitated products showed higher digestibility and foam stability. Membrane processing can therefore reduce aggregation associated with isoelectric precipitation, but the purity of the final product still depends strongly on the composition of the feed stream and the pretreatment applied before filtration.

Diafiltration water requirements and membrane fouling are important considerations during membrane concentration. Rizki et al. [95] developed a model for a four-stage ultrafiltration–diafiltration process and showed that recycling selected process streams could reduce diafiltration liquid requirements by up to 79% while increasing product purity and dry matter content. Proteins, polysaccharides, lipids, and their aggregates can accumulate on the membrane surface and form a resistant fouling layer. Alpiger et al. [96] found that irreversible fouling accounted for more than 68% of the total resistance during filtration through 10 and 100 kDa membranes. Pectinase treatment during extraction reduced irreversible fouling of the 10 kDa membrane to approximately 40% and increased carbohydrate transmission into the permeate. The performance of the membrane is strongly influenced by the colloidal composition of the protein extract, while enzymatic degradation of co-extracted polysaccharides can improve membrane flux and facilitate impurity removal.

Microfiltration can be introduced before ultrafiltration to remove oil droplets and larger colloidal particles. Chandrappa et al. [97] treated aqueous rapeseed protein-rich extracts using hydrophilic silicon carbide ceramic membranes. Approximately 98.7–99% of the oil was retained, while most proteins entered the low-oil permeate intended for subsequent concentration. Voudouris et al. [98] used a 5 kDa membrane to refine a mildly extracted rapeseed protein stream. Membrane filtration increased the protein content of the dried product from 45.1% to 63.4% while removing part of the phenolic compounds, carbohydrates, and minerals. The refined proteins also formed stronger interfacial films and more stable foams. Membrane processing can also be combined with selective extraction to recover a specific protein fraction. Albe-Slabi et al. [99] selectively extracted napin from cold-pressed rapeseed meal under acidic saline conditions, obtaining a napin extraction yield above 55% and a napin proportion above 95% in the extract. Subsequent ultrafiltration produced light-colored napin-rich fractions with high solubility and preserved foaming and emulsifying properties. This approach extends membrane processing from the concentration of mixed protein extracts to the purification of selectively extracted protein fractions.

When higher-purity protein fractions are required, chromatography offers greater selectivity than membrane separation. Bérot et al. [100] combined ion-exchange, size-exclusion, and hydrophobic-interaction chromatography to process 3.5 kg of rapeseed meal, obtaining cruciferin, napin, and lipid transfer protein fractions with purities above 95%. However, the recoveries of cruciferin and napin were only approximately 40% and 18%, respectively, while the multistep procedure also required considerable buffer consumption and operational complexity. To simplify the multistep chromatographic workflow, Moreno-González et al. [101] screened eight mixed-mode and ion-exchange resins using a high-throughput platform. POROS 50HS showed selective binding toward napin, and subsequent model-based optimization predicted purities and recoveries above 98% for both napin and cruciferin. Building on this selective binding behavior, Arnecke et al. [102] coupled cation exchange capture of napin with anion-exchange recovery of cruciferin in a continuous multicolumn process, thereby limiting intermediate handling and delays between the two separation stages. These developments illustrate how chromatographic purification has moved toward more selective resin selection, model-assisted optimization, and continuous processing.

Beyond separating individual protein fractions, downstream purification may also focus on removing specific low molecular weight compounds. Ayan et al. [103] used charged membranes under an applied electric field to promote the migration of sinapic acid while retaining rapeseed proteins. In a larger system equipped with five anion exchange membranes, 90.3% of the sinapic acid was removed within 240 min, with 88.8% of the protein retained. This method limits direct exposure of the proteins to organic solvents, although its performance remains sensitive to membrane charge, ionic strength, treatment time, and protein–phenolic interactions. It is therefore better suited to final product polishing after protein concentration than to primary protein recovery.

Precipitation and membrane processing are suited to bulk protein recovery and compositional adjustment, whereas chromatography and electrically assisted separation provide the additional selectivity needed for individual protein fractions or targeted contaminant removal. An effective downstream process should combine these operations according to the required balance among protein recovery, purity, functionality, and processing demand.

### 3.4. Fractionation of Phenolic Compounds, GLS, and Phytic Acid

#### 3.4.1. Recovery of Phenolic Compounds

Defatted rapeseed meal still contains appreciable amounts of phenolic compounds, predominantly sinapine, together with sinapic acid and its derivatives [104]. These phenolic compounds can be recovered as valuable components (large-scale utilization of purified rapeseed meal-derived phenolic fractions has not yet been established; see Section 5), but they may also be transferred into the aqueous phase during protein extraction and adversely affect the color and flavor of the resulting protein products [105]. Current studies have mainly focused on the direct extraction of phenolic compounds from solid rapeseed meal and their recovery from aqueous side streams generated during protein processing, followed by selective enrichment and purification [106].

When phenolic compounds are recovered directly from solid rapeseed meal, solvent composition, particularly the proportion of water in the extraction mixture, is an important factor affecting extraction efficiency. Aqueous ethanol has subsequently been used as a practical solvent system for rapeseed meal phenolics, with extraction efficiency further improved by increasing temperature and pressure. Nandasiri et al. [107] employed accelerated solvent extraction with 70% ethanol at 1500 psi and 180 °C, achieving a total phenolic yield of 24.71 mg sinapic acid equivalents (SAE)/g DM. However, intensifying extraction conditions can also modify the recovered phenolic profile rather than simply increase recovery. Li and Guo [108] showed that elevated temperatures and alkaline conditions also promoted the conversion of sinapine into sinapic acid and canolol (4-vinylsyringol); thus, increased extraction yield does not necessarily indicate that the original phenolic composition has been fully preserved. Another intensification strategy is ultrasound-assisted extraction. Ding et al. [109] introduced nitrogen during ultrasonication to reduce free-radical formation and the associated oxidative reactions, achieving a polyphenol yield of 12.64 mg/g under the optimized conditions. In addition to conventional alcoholic solvents, deep eutectic solvents [110] have also been investigated for the extraction of phenolic compounds from rapeseed meal. Wongsirichot et al. [111] reported a maximum sinapic acid extraction yield of 85.69% using a deep eutectic solvent (DES) system, exceeding the 59.54% obtained with methanol in the same study. However, additional separation is required to recover the phenolic compounds from the DES phase.

In addition to the direct treatment of solid rapeseed meal, aqueous side streams generated during protein isolation can serve as feedstocks for phenolic recovery. Because sinapine and other sinapic acid derivatives have relatively low molecular weights, they predominantly pass into the permeate during the ultrafiltration concentration of proteins, thereby achieving an initial separation from the membrane-retained protein fraction [112]. These side streams generally contain several esterified sinapic acid derivatives, and the subsequent processing route can therefore be selected according to the intended product. When sinapic acid is the target compound, sinapine and other esterified derivatives can first be hydrolyzed into sinapic acid, followed by adsorption-based enrichment. By combining hydrolysis with adsorptive separation, Thiel et al. [113] enriched sinapic acid by approximately 4.5- to 5.1-fold. When the retention of sinapine is desired, complete hydrolysis should be avoided. Instead, ion-exchange separation can exploit the quaternary ammonium group of sinapine, which remains positively charged in aqueous solution. Kdah et al. [114] adjusted rapeseed protein production effluent to pH 4 and adsorbed sinapine using the weak cation exchange resin C106, which contains carboxylic-acid exchange sites. The resin was first eluted with 50% ethanol to remove sinapic acid and other neutral derivatives retained predominantly through hydrophobic interactions. Acidified ethanol was subsequently applied to weaken the ionic interactions between sinapine and the resin, ultimately yielding a sinapine fraction with purity of 98.85%.

For the recovery of target phenolic compounds from crude extracts or protein-processing side streams, adsorptive separation provides greater selectivity than simple concentration. Macroporous resins adsorb phenolic compounds primarily through hydrophobic interactions with the polymeric resin matrix, whereas more hydrophilic components, including sugars, phytic acid, and GLS, are preferentially retained in the aqueous phase. Among the resins screened by Moreno-González, Girish, Keulen, Wijngaard, Lauteslager, Ferreira, and Ottens [105], the food-grade FPX66 resin exhibited maximum sinapic acid adsorption capacity of 102.6 mg/g and allowed desorption with 70% ethanol. It also showed high selectivity for sinapic acid over glucose, phytic acid, and GLS. Building on these findings, adsorption was subsequently extended from conventional batch packed-bed columns to a semi-continuous CaptureSMB process. At a yield above 97%, productivity increased from 5.18 to 10.3 g/L/h, demonstrating that the continuous switching of adsorption columns can improve resin utilization and processing capacity [115]. When the intended product is a mixed phenolic-rich fraction rather than a highly purified compound, acidified ethanol extraction combined with AB-8 resin purification can increase the total phenolic content of rapeseed meal extracts to 406.63 mg SAE/g [116]. In contrast, molecularly imprinted polymers (MIPs) have been investigated for the selective recovery of individual phenolic compounds. Sinapic acid-selective MIPs showed adsorption capacities of 141.3–154 mg/g, while the adsorption equilibrium time was reduced from 60 min in the earlier material to 10 min in a subsequent metal–organic framework (MOF)-composite system [117,118]. These studies demonstrate the potential of MIPs for selective sinapic acid recovery, although their application has so far remained primarily at the laboratory scale.

Integrating the phenolic recovery into the fractionation and biorefining of rapeseed meal can reduce the loss of low-molecular-weight compounds through liquid side streams and improve the overall utilization of the raw material. Its practical implementation will depend on solvent and adsorbent recycling, the maintenance of separation performance under complex feed conditions, and the consistency of product composition during continuous operation.

#### 3.4.2. Removal, Recovery, and Conversion of GLS

GLS are major antinutritional constituents of rapeseed meal. GLS and several GLS hydrolysis products contribute to bitterness, reduced palatability, and adverse physiological effects, thereby limiting the use of rapeseed meal as a protein-rich ingredient in food and feed [119]. From the valorization perspective, intact GLS can also be recovered as precursors for bioactive isothiocyanates with potential applications in food products, nutraceutical formulations, and crop protection [120]. The high water solubility of GLS facilitates GLS transfer from the solid matrix into aqueous fractions during processing [121]. Existing treatments for GLS include aqueous and hydroalcoholic extraction, removal during protein fractionation, enzymatic conversion, and microbial fermentation.

Direct extraction of GLS serves two purposes in rapeseed processing: reducing GLS in the solid material and recovering intact GLS from the liquid extract. Fauduet et al. [122] treated dehulled rapeseed meal twice with water at pilot scale and also removed approximately 98% of GLS, although 30% of the initial dry matter and almost 25% of the crude protein entered the aqueous extract. The aqueous leaching can remove most of the GLS, but the concurrent loss of dry matter and protein limits process selectivity; the resulting GLS-rich liquid stream may nevertheless be further processed for GLS recovery. Further enrichment can be achieved by chromatographic purification. Ishikawa et al. [123] directly extracted rapeseed meal with 20% ethanol and increased the GLS content from 3.0% in the crude extract to 39.8% after silica gel chromatography.

Recovery of intact GLS from cold-pressed rapeseed meal requires suppression of residual myrosinase, because uncontrolled GLS hydrolysis can generate toxic nitriles and goitrogenic goitrin [119]. Albe-Slabi et al. [15] found that water or ethanol concentrations below 40% caused 90–95% GLS degradation. Extraction with 75% ethanol at 60 °C inhibited myrosinase activity, and three extraction stages recovered approximately 75% of intact GLS while reducing the residual GLS content of the protein-rich solid to below 4 μmol/g. GLS extraction can also be introduced before meal production. Citeau et al. [14] used aqueous ethanol or isopropanol as oil-extraction solvents for whole rapeseed; 84% isopropanol reduced the GLS concentration of the resulting meal by 49–73% compared with the other alcohol systems. In a different front-end process, Dumpler et al. [120] extracted whole dehulled rapeseed kernels with natural deep eutectic solvents (NADES) before further seed fractionation. A betaine–1,2-propanediol-based NADES containing 20% water extracted up to 3.48 g/kg GLS, whereas increasing the water content to 30–40% lowered intact GLS recovery because of endogenous myrosinase activity. Beyond dedicated GLS extraction, aqueous protein fractionation also transfers GLS into supernatants and membrane permeates.

During aqueous fractionation, the reduction in GLS content is mainly attributable to dissolution into the aqueous phase, membrane permeation, retention in the supernatant after acid precipitation, and hydrolysis catalyzed by residual myrosinase. Tzeng et al. [92] extracted defatted rapeseed meal at pH 10.5–12.5, followed by isoelectric precipitation, ultrafiltration, and diafiltration of the acid-soluble liquid phase. GLS were undetectable in all fractions obtained from the process. Ser et al. [121] further established a GLS mass balance during ultrafiltration. When the Amicon stirred ultrafiltration cell fitted with a 10 kDa molecular weight cut-off membrane was used, 63% of the initial GLS entered the discarded permeate. This proportion decreased to 39% when a Vivaflow 200 tangential-flow ultrafiltration system equipped with a 30 kDa membrane was applied. The final precipitated fractions obtained using both processes retained only approximately 1.9% of the initial GLS, indicating that GLS partitioning is also influenced by membrane-module configuration and filtration mode. Kalaydzhiev et al. [90] sequentially washed rapeseed meal four times with 75% ethanol at a solid loading of 25% (*w*/*v*), with each washing step lasting 30 min. The washed residue was subsequently extracted at pH 12 and 40 °C for 60 min. Two stepwise precipitation routes were applied, involving a gradual decrease in pH from 10.5 to 2.5 or a gradual increase from pH 2.5 to 8.5. GLS were undetectable in all final fractions obtained through these routes. Ahlström et al. [88] extracted cold-pressed rapeseed meal at pH 10.5 and adjusted the liquid phase to pH 3.5 for precipitation. The GLS content decreased from 16 μmol/g in the raw material to 0.12 μmol/g in the fraction obtained after the first precipitation cycle and fell below the detection limit in the fraction obtained after the second cycle. These results show that water-soluble GLS remained predominantly in the supernatant after acid precipitation. Du et al. [17] treated rapeseed meal with 1% of a multienzyme preparation at a solid-to-liquid ratio of 1:10 and 40 °C for 4 h, followed by extraction for an additional 1 h with NaOH added at 4% of the rapeseed meal mass. The GLS contents of the fractions obtained after enzymatic treatment and combined enzyme–alkali treatment were significantly lower than that of the fraction produced by alkali extraction alone. Enzymatic conversion may also contribute to the decrease in GLS concentration. Miklavčič Višnjevec et al. [124] found that residual myrosinase rapidly hydrolyzed GLS when water was added to cold-pressed rapeseed meal at its native pH of approximately 5. Following treatment by 0.2 μm microfiltration and 5 kDa ultrafiltration, intact GLS in the final fraction were below the detection limit, whereas the goitrin content reached 95 mg/kg. In two processing routes combining 0.2 μm microfiltration with 10 kDa ultrafiltration, the goitrin contents were 7 and 22 mg/kg, respectively. Accordingly, the GLS content of the final fraction should be interpreted together with measurements of GLS in the permeate and precipitation supernatant, as well as the major GLS hydrolysis products, to account for both physical partitioning and enzymatic degradation.

Beyond the physical partitioning of GLS through extraction and membrane separation, enzymatic and oxidative treatments can directly degrade GLS and related harmful products in rapeseed meal or its processing streams. Xie et al. [125] directly added myrosinase derived from rapeseed sprouts to rapeseed meal; then, a 4 h reaction with 2236.35 U/g myrosinase, 9.63 μg/g ascorbic acid, and 26.68 μg/g ethylenediaminetetraacetic acid resulted in the degradation of more than 80% of the GLS. The treatment also produced 859.30 μg/g isothiocyanates and 685.59 μg/g oxazolidine-2-thione derivatives, showing that myrosinase treatment reduces intact GLS while generating multiple hydrolysis products. To degrade the toxic nitriles formed through GLS hydrolysis in rapeseed meal, Zhang et al. [126] applied BnNIT2 (nitrilase derived from *Brassica napus*) to 3-hydroxy-4-pentenenitrile, 3-butenenitrile, and 4-pentenenitrile, reducing the total nitrile content by approximately 80%. For rapeseed meal GLS transferred into the aqueous phase, Yan et al. [127] applied electrochemical advanced oxidation using a boron-doped diamond anode. Treatment for 120 min at a flow rate of 400 mL/min, a current density of 7.75 mA/cm^2^, and a NaNO_3_ concentration of 2.0 mM achieved 82.2% GLS mineralization and reduced the biological toxicity of the treated liquid. However, enzymatic treatment generally requires the preparation or addition of specific enzymes and depends on appropriate pH, temperature, cofactors, and reaction time. Myrosinase converts GLS into hydrolysis products with different chemical profiles, including isothiocyanates, nitriles, and oxazolidine-2-thiones. Some of these products retain potential toxicity and require further detoxification and monitoring, increasing the complexity of process control. Electrochemical advanced oxidation is mainly applicable to processing streams in which GLS have already entered the aqueous phase. Its implementation requires specialized electrodes and reaction equipment, together with electrical energy and supporting electrolytes. Electrode cost, electrolyte addition, mass-transfer efficiency, and interference from coexisting organic constituents in complex liquid streams also require consideration during process scale-up. Enzymatic treatment and electrochemical oxidation are therefore most applicable as targeted detoxification operations at selected processing stages. Microbial fermentation provides a broader treatment route by continuously degrading GLS through microbial growth and in situ enzyme production while simultaneously transforming several antinutritional factors, and has been more widely investigated for the detoxification of rapeseed meal.

Microbial fermentation enables continuous GLS degradation through enzyme systems produced in situ by microorganisms growing in rapeseed meal, thereby reducing reliance on externally added purified enzymes. Lactic acid bacteria and Bacillus spp. are among the microorganisms most commonly used for this purpose. Lactic acid bacteria are well suited to food and feed fermentation, while Bacillus spp. generally possess strong extracellular enzyme-secretion capacity and adaptability to solid-state fermentation. The Lactiplantibacillus strains screened by Chen et al. [128] reduced total GLS by approximately 80%. Zhang et al. [129] achieved a GLS degradation rate of 94.62% using a coculture of *Lactobacillus delbrueckii* and *Bacillus subtilis*. Filamentous fungi can secrete cellulases, hemicellulases, and related enzymes that disrupt the cell-wall matrix of rapeseed meal and increase the accessibility of tissue-entrapped GLS to degradative enzyme systems. *Rhizopus oligosporus*, *Trichoderma reesei*, *Pleurotus ostreatus*, and *Aspergillus* spp. have all shown GLS-degrading capacity, with degradation rates above 95% reported in some studies [130,131,132,133,134]. The combined use of different microorganisms can further exploit the complementarity of microbial enzyme systems and metabolic pathways. Mixed cultures combining Bacillus spp., lactic acid bacteria, and yeasts have also been applied to rapeseed meal fermentation [135]. Hong et al. [136] constructed a four-species microbial consortium that reduced GLS by 86.08% and simultaneously decreased 5-vinyl-1,3-oxazolidine-2-thione. The advantages of microbial fermentation arise from direct microbial degradation of GLS, cell-wall deconstruction, and complementary metabolic activities. Treatment duration and degradation efficiency remain dependent on strain composition and fermentation conditions.

#### 3.4.3. Removal and Recovery of Phytic Acid

Phytic acid is a major phosphorus-containing antinutritional factor in rapeseed meal. Chemically, phytic acid is myo-inositol hexakisphosphate (InsP_6_) and occurs predominantly as phytate salts in the raw material. Rapeseed meal generally contains approximately 2–5% phytic acid, although the reported concentration varies with cultivar, raw material type, and analytical method [137]. Microscopic observations have shown that phytate is mainly stored as phytate globoids within protein storage structures in seed cells [138]. This spatial association facilitates the co-migration of phytate with storage proteins during protein fractionation. Serraino and Thompson [139] found that the association between phytic acid and rapeseed proteins was affected by pH and mineral ions. The multiple phosphate groups of phytic acid can also form complexes with Ca^2+^, Mg^2+^, Zn^2+^, and Fe^2+^/Fe^3+^, thereby affecting mineral bioavailability as well as protein solubility and digestibility.

During rapeseed protein fractionation, the distribution of phytic acid depends on protein solubilization and precipitation, the stability of phytic acid–protein complexes, and the subsequent purification process. Tzeng et al. [92] found that part of the phytic acid could be removed during ultrafiltration and diafiltration, whereas phytic acid associated with proteins could be retained by the membrane together with macromolecular components. Subsequent anion-exchange treatment further reduced residual phytic acid in the protein products [140]. Direct comparisons of extraction methods have also shown that the salt-extracted protein prepared by Li et al. [63] contained 0.59% phytic acid, which was lower than the level in the alkali-extracted protein. By contrast, Đermanović et al. [78] found that protein isolates obtained using deep eutectic solvents contained more phytic acid than the corresponding alkaline extract. Increased protein yield or purity therefore does not necessarily result in lower phytic acid content. The final phytic acid concentration also depends on the ability of the extraction system to weaken the interactions of phytic acid with proteins and mineral ions.

Phytic acid may also be recovered as a phosphorus-containing fraction rather than being treated solely as an antinutritional factor requiring removal. Pan et al. [141] extracted phytic acid from double-low cold-pressed rapeseed meal using acetic acid and recovered the proteins present in the crude extract through membrane separation. Phytic acid was not detected in the resulting protein fraction. Thiel et al. [113] separated phytic acid from rapeseed meal extracts using an anion-exchange resin. These studies integrated phytic acid extraction with protein recovery or the fractionation of processing streams, providing feasible routes for the recovery and subsequent utilization of phytic acid.

When phytic acid remains tightly associated with proteins or persists in protein products, phytase hydrolysis can directly degrade the inositol phosphate structure. Kasprowicz-Potocka et al. [142] evaluated several commercial phytase and carbohydrase preparations during solid-state enzymatic treatment of rapeseed meal. Phytase-containing treatments reduced phytate more effectively than carbohydrase-only treatments, while combining carbohydrases with phytase did not provide further improvement compared with phytase alone. Maenz et al. [143] further demonstrated that complexes formed between phytic acid and mineral ions such as Zn^2+^ and Fe^2+^ reduced the accessibility of phytic acid to phytase, while the addition of chelating agents such as citric acid promoted phytate hydrolysis. Phytase treatment can also improve subsequent protein recovery. Rodrigues et al. [67] found that phytase pretreatment increased protein extraction under alkaline conditions and reduced the phytic acid concentration in the resulting protein concentrate to approximately 1 g/kg. The effectiveness of phytase treatment is therefore determined by enzyme dosage, reaction pH, auxiliary enzymatic activities, and the mineral-binding state of phytic acid.

Microbial fermentation relies on the in situ secretion of phytase and other hydrolytic enzymes and can reduce phytic acid while modifying the protein and fiber structures of rapeseed meal. Solid-state fermentation can simultaneously reduce phytic acid and other antinutritional components in rapeseed meal. Olukomaiya et al. [144] fermented canola meal with *Aspergillus sojae*, *A. ficuum*, and their co-cultures and observed significant reductions in both phytic acid and total GLS, together with changes in protein molecular distribution and functional properties. Zhu et al. [145] further used a mixed culture of *Bacillus subtilis*, *Saccharomyces cerevisiae*, and *Bacillus amyloliquefaciens* for solid-state fermentation of rapeseed meal. Phytic acid, GLS, crude fiber, and tannins were reduced by 42.41%, 99.18%, 27.21%, and 34.17%, respectively, while crude protein, amino acids, and peptides increased after fermentation. These results show that fermentation can reduce phytic acid together with other antinutritional factors, although the extent of degradation depends on the microorganisms used and the fermentation conditions.

### 3.5. Comparative Assessment of Fractionation and Recovery Strategies

The selection of an appropriate fractionation strategy for rapeseed meal depends primarily on the degree of refinement required for the intended product. Milling–sieving, air classification, and electrostatic separation mainly redistribute proteins and fiber according to differences in particle properties and therefore generally produce enriched rather than highly purified fractions. Wet fractionation provides greater opportunities for releasing proteins, polysaccharides, and other bound components and for obtaining products of higher purity through subsequent purification. These approaches consequently serve different processing objectives. For feed, composite food ingredients, and some material applications where multicomponent fractions are acceptable, dry fractionation can reduce the need for water, chemicals, concentration, and drying. Higher-purity products, by comparison, generally require aqueous extraction and additional purification. Studies on plant protein processing have similarly shown that increasing the degree of refinement is commonly associated with greater resource inputs, while the functional requirements of the final application determine whether this additional refinement is justified [146,147,148]. In dry fractionation, further improvements in separation efficiency are also constrained by the extent of tissue disintegration. Wockenfuss et al. showed that reprocessing the coarse fraction recovered part of the protein that had already been liberated but was incorrectly allocated during the first separation, whereas further treatment of the already enriched fine fraction produced only limited compositional changes [34]. Under these conditions, improving the liberation of components becomes more effective than simply increasing the number of classification steps. The main differences among these fractionation and recovery strategies in terms of yield, selectivity, product functionality, resource demand, scalability, side-stream generation, and downstream compatibility are summarized in Table 1.

Wet processing provides greater flexibility for component release and purification, while placing stronger emphasis on the balance between product quality and processing intensity. For proteins, intensive alkaline extraction can increase recovery, although high pH also affects protein conformation, color, and the composition of co-extracted compounds. Salt extraction, enzyme-assisted extraction, and DES/NADES systems operate under different chemical environments and can therefore produce proteins with different compositions, structures, and functional properties. Similar differences arise during downstream recovery. Isoelectric precipitation preferentially recovers cruciferin, whereas a substantial proportion of napin remains in the acidic supernatant, and the functional properties of the precipitated proteins vary with precipitation pH. Ahlström et al. found that the conditions providing higher protein recovery did not coincide with those yielding the most stable emulsions [88]. Protein composition and functionality therefore provide an important basis for selecting processing conditions together with recovery yield. Karimi et al. reported a related trade-off for NADES extraction: protein recovery was lower than that obtained under intensive alkaline conditions, while the resulting proteins showed advantages in color, structural preservation, and some functional properties [77]. The value of such milder extraction systems thus lies particularly in product quality and selectivity. Resource demand in wet processing also extends well beyond the extraction step. Aqueous streams subsequently require solid–liquid separation, desalting, concentration, and drying. Life-cycle assessment by Lie-Piang et al. showed that a higher degree of protein refinement increased environmental impacts, with drying representing an important contribution in several impact categories [146]. Although that analysis covered plant materials beyond rapeseed meal, it highlights a relevant process consideration: the total liquid volume handled and the subsequent water-removal requirement provide a more representative indication of processing intensity than extraction temperature or treatment time alone. Process design can substantially influence these demands. Rizki et al. reduced diafiltration liquid requirements by up to 79% through stream recycling in a multistage UF/DF process [95], while the eREX-based process developed by Ghosh et al. combined intensified pretreatment with a shorter alkaline extraction and reduced water use, energy consumption, and production cost [68].

From the perspective of whole-meal utilization, the way individual operations are connected becomes particularly important. During protein extraction, phenolic compounds, GLS, phytate, and low-molecular-weight carbohydrates are redistributed into precipitation supernatants or membrane permeates. These streams can serve as feedstocks for further recovery rather than remaining solely as process effluents. Le et al., for example, recovered phenolic compounds directly from a by-product stream generated during rapeseed protein isolate production [112], while Thiel et al. integrated the recovery of proteins, sinapic acid, and phytic acid within the same downstream processing scheme [113]. Such approaches increase the value of purification by converting compounds removed from the principal product into additional co-products. The efficiency of a separation process can therefore be assessed more comprehensively by considering the concentration and recoverability of components in the resulting side streams together with the purity of the main product. Sequential valorization extends this concept further. Wongsirichot et al. recovered valuable components before using the remaining rapeseed meal for biological conversion [111], whereas Li and Guo produced antioxidants, proteins, and monosaccharides sequentially from the same raw material [151]. In these systems, processing order directly influences the condition of the feed entering the next stage. A preceding treatment may improve accessibility of residual components, while changes in pH, salt content, thermal history, or molecular structure can alter their subsequent processing behavior. Compatibility between consecutive operations is therefore central to the design of an effective cascade process.

Overall, rapeseed meal fractionation is better guided by the minimum degree of refinement required for the intended application. Food-grade protein ingredients generally require tighter control of color, flavor, antinutritional factors, and functionality and can therefore justify more extensive purification. Feed, fermentation, material, and thermochemical applications can often accommodate fractions or residues with more complex compositions. Matching fractions of different purity to appropriate applications can reduce unnecessary processing of lower-value components. Future comparisons of fractionation technologies should therefore place greater emphasis on complete mass balances and on the final destinations of the main product, co-products, liquid side streams, and solid residues, together with associated water, chemical, energy, concentration, and drying requirements. The amount of usable product generated from a given quantity of raw material, relative to the net processing inputs required, provides a more meaningful measure of the practical competitiveness of a fractionation route than the maximum recovery of a single component.

## 4. Current Utilization Routes for Rapeseed Meal

### 4.1. Feed and Fertilizer

Rapeseed meal is most commonly used as a protein source in animal feed [12]. It contains a relatively high level of protein and is rich in sulfur-containing amino acids, although its available energy content and the availability of several amino acids are generally lower than those of soybean meal [155]. This difference is primarily attributable to hull-derived fiber and cell-wall non-starch polysaccharides, which are only partially degraded in the digestive tract and consequently restrict nutrient release and energy utilization [9]. The adverse effects of structural carbohydrates are more pronounced in monogastric animals because a substantial proportion of the recalcitrant fiber in rapeseed meal remains poorly utilized [156]. GLS and their hydrolysis products, phytate, sinapine, and other phenolic compounds further reduce palatability, mineral availability, and protein digestion [157]. Moderate desolventizing and toasting help remove residual solvent and reduce some heat-sensitive antinutritional factors, whereas excessive thermal exposure decreases reactive lysine content and overall protein quality [6]. To alleviate the limitations associated with fiber, milling, sieving, and air classification can redistribute protein and hull-rich fiber components, thereby producing fractions with higher protein contents and improved digestibility [8]. Fermentation can further degrade selected antinutritional factors and macromolecular components, improve protein and amino acid utilization, and support intestinal health in monogastric animals [158]. The feeding value of rapeseed meal therefore depends on both raw-material composition and processing history, and an appropriate inclusion level should be determined according to fiber content, antinutritional factor levels, and the digestive capacity of the target animals.

In addition to feed use, rapeseed meal can be applied as organic fertilizers to return residual organic matter and mineral nutrients to agricultural systems [12]. Fermentation can convert part of the protein into free amino acids and short peptides while reducing the contents of GLS hydrolysis products such as isothiocyanates and oxazolidine-2-thiones, thereby improving nutrient availability and application safety [159]. The incorporation of fermented rapeseed meal into soil can increase organic matter, available nitrogen, and available phosphorus, enhance soil enzyme activities associated with nutrient cycling, and modify the composition of the rhizosphere microbial community [160]. In addition to nutrient supply and microbial regulation, bioactive compounds formed from residual GLS can suppress certain plant-parasitic nematodes, giving rapeseed meal potential as a biofumigant [161]. This suppressive effect is governed by dosage and timing because excessive application rates or sowing immediately after application may also inhibit seed germination and seedling growth [161]. The use of rapeseed meal as a fertilizer or soil amendment therefore requires fermentation or composting to promote nutrient conversion, together with appropriate control of the application rate and the interval between soil incorporation and sowing.

### 4.2. Applications of Rapeseed Meal Protein

#### 4.2.1. Food Applications

Protein ingredients commonly contribute to interfacial stabilization, water retention, and structure formation in foods, and the applications of rapeseed protein have largely been developed around these functions [137]. Among the reported applications, emulsions and lipid structuring have been studied relatively systematically, covering protein adsorption at oil–water interfaces, droplet stabilization, gel-like emulsions, oleogels, and the subsequent use of these systems in food products [162]. Tang and Ghosh [163] used rapeseed protein isolate to prepare concentrated oil-in-water emulsions with an oil volume fraction of 50%. Increasing the protein concentration reduced droplet size and enhanced the viscoelasticity of the system, while ionic strength, acidification, and heat treatment further affected droplet aggregation and emulsion stability. Xu et al. [164] subsequently constructed gel-like high internal phase emulsions through a protein aggregation network in the continuous phase, and the resulting emulsions exhibited a certain degree of freeze–thaw stability. Cai et al. [165] directly used salt-extracted and ultrafiltered rapeseed protein to stabilize algae oil high internal phase emulsions, improving the oxidative stability of the algae oil and the lubrication properties of the emulsions while maintaining their viscoelastic structure. Tang and Ghosh [166] also prepared emulsion-templated oleogels through heat-induced aggregation of interfacial proteins and used the resulting oleogels as shortening replacers in cakes, thereby extending the interfacial stabilization function of rapeseed protein to semisolid lipid structuring and bakery applications.

In solid foods requiring the formation of continuous structures, rapeseed protein participates in processing together with other proteins, starch, and water; the structuring performance depends on protein composition and blending ratio. Jia et al. [167] subjected rapeseed protein concentrate, either alone or blended with wheat gluten, to high-temperature shear processing. Rapeseed protein concentrate formed a partially fibrous structure, while the addition of wheat gluten enhanced structural anisotropy.

Bakery applications have been examined with studies covering dough processing behavior, final product quality, and storage stability. Witczak et al. [168] replaced part of the starch in gluten-free formulations with rapeseed protein isolate. Addition levels of 6–15% altered dough viscoelasticity, flow behavior, and pasting properties. Korus et al. [169] further evaluated gluten-free bread within the same addition range. Rapeseed protein increased the protein content of the finished bread and affected loaf volume, crumb structure, color, hardness, and sensory quality. The nutritional enhancement achieved at higher addition levels was generally accompanied by more pronounced structural and sensory changes. Wang et al. [170] fermented rapeseed protein ingredients with lactic acid bacteria, with dextran formed in situ during fermentation. When the fermented protein ingredients were incorporated into wheat bread, loaf-specific volume increased, crumb hardness decreased, and the contents of several free amino acids increased.

Rapeseed protein has also been used to improve the nutritional composition of foods intended for specific population groups. Di Lena et al. [171] incorporated rapeseed meal protein isolate into texture-modified chicken, vegetable, and bread products for older adults, increasing the protein and selected mineral contents of the products. The effects of protein addition on color, odor, texture, and overall acceptability varied with the food matrix and inclusion level. The appropriate inclusion level of a given rapeseed protein ingredient may differ among food products.

The nutritional suitability of refined rapeseed protein as a human food protein source is also supported by human studies. Using stable-isotope tracing, Bos et al. [172] found that rapeseed protein had a relatively low true ileal digestibility but a high metabolic utilization of absorbed amino acids. Fleddermann et al. [173] compared rapeseed protein isolate, rapeseed protein hydrolysate, and soy protein in a randomized crossover trial and evaluated the nutritional quality of the protein preparations based on plasma amino acid responses and nitrogen balance. Volk et al. [174] further investigated postprandial metabolic responses to rapeseed protein in healthy participants. Most metabolic responses to rapeseed and soy proteins were comparable, whereas rapeseed protein produced a lower postprandial insulin response and greater satiety. However, the refined rapeseed protein preparations generally contain more protein and less fiber and residual antinutritional compounds than less refined protein concentrates, which may lead to different nutritional responses.

The use of rapeseed protein in liquid foods remains constrained by limited solubility and susceptibility to heat-induced aggregation. Limited hydrolysis, glycation, high-pressure homogenization, adjustment of pH and ionic conditions, and incorporation of hydrocolloids can improve the solubility, interfacial properties, and colloidal stability of rapeseed protein [175]. However, validation in complete ready-to-drink beverage formulations subjected to pasteurization or high-temperature treatment and long-term storage remains limited.

In addition to serving directly as a nutritional and structuring ingredient, rapeseed protein has begun to be investigated as a carrier for bioactive compounds. Aktaş et al. [176] used rapeseed protein isolate as an interfacial material in double emulsions for anthocyanin encapsulation and compared its performance with protein isolates from black caraway cake and wheat bran. Canola protein formed anthocyanin-containing encapsulation systems, although droplet uniformity, encapsulation efficiency, and emulsion stability were affected by protein concentration and emulsification conditions. In a subsequent study, the double emulsions were dried to produce microcapsules, and the effects of canola, black caraway, and soy proteins on microcapsule structure, anthocyanin retention, solubility, and antioxidant activity were compared [177]. These studies expand the potential use of rapeseed protein as a delivery material, although the available evidence is still largely based on model bioactive compounds and laboratory-scale preparation systems.

Color and flavor directly influence the amount of rapeseed protein that can be incorporated into food products. Different extraction methods alter the color, odor, and co-extracted components of protein preparations, and proteins obtained by weakly acidic salt extraction generally exhibit a lighter color and milder odor [62]. Chen et al. [178] sequentially treated rapeseed protein isolate with phytase and ethanol, reducing several volatile compounds associated with sour and pungent odors while increasing esters associated with fruity and sweet aromas. Asen et al. [179] refined canola protein isolate using XAD-16N, HP-20, and SP-207 adsorption resins. Resin treatment reduced sinapine, GLS, pigments, and several volatile off-flavor compounds while improving protein color and solubility. Flavor improvements achieved at the ingredient level still require confirmation through sensory evaluation and storage studies in specific food products.

#### 4.2.2. Material and Other Non-Food Applications

Beyond the use as food ingredients, the application of rapeseed proteins in materials and other non-food systems can broaden the utilization pathways of protein recovered from rapeseed meal and provide additional uses for protein fractions differing in purity and composition. These applications rely mainly on protein structural adaptability and interfacial functionality and are less dependent on nutritional composition and sensory quality. Rapeseed proteins exhibit film-forming, adhesive, self-assembling, water-absorbing, and interfacial properties, while their functional groups can participate in hydrogen bonding, hydrophobic interactions, and covalent cross-linking [56,180]. These characteristics support their use in protein-based plastics and films, packaging coatings, wood adhesives, nanodelivery carriers, hydrogels, and surface-active systems.

The formation of protein-based films depends on the rearrangement of protein chains into continuous networks during plasticization, denaturation, and drying. Jang et al. [181] prepared rapeseed protein films using different plasticizers and emulsifiers and found that tensile properties and extensibility varied with formulation. Chang and Nickerson further examined the effects of plasticizer type, genipin cross-linking, and protein and glycerol concentrations [182,183]. Genipin increased tensile strength in some plasticized systems, whereas increasing the glycerol concentration improved film flexibility but reduced strength and increased water-vapor permeability. Shi and Dumont [184] prepared rapeseed protein isolate films by glycerol plasticization and solution casting and adjusted their mechanical properties and water absorption through sodium dodecyl sulfate-induced denaturation and stearic acid incorporation.

The composition of the protein material and the isolation method also affect material performance. Fetzer et al. [185] compared protein concentrates prepared from different rapeseed press meals by acid precipitation and ultrafiltration. All concentrates formed continuous films, although protein content, solubility, film strength, and extensibility varied with the starting material and isolation route. Chemical modification has mainly been used to strengthen interactions between protein chains or reduce material hydrophilicity. Li et al. [186] used a diepoxy compound to promote covalent cross-linking between rapeseed protein chains and prepared films by wet casting followed by hot pressing. Cross-linking increased thermal stability and tensile performance and reduced phase separation between cruciferin and napin. He et al. [187] succinylated rapeseed protein isolate. A low degree of succinylation improved the mechanical and barrier properties of the films, whereas further modification did not produce a proportional improvement. Fetzer et al. [188] modified rapeseed protein concentrates with lauroyl chloride and oleoyl chloride. Long-chain acylation reduced protein water solubility and film surface energy and increased tensile strength, but did not fully resolve the poor stability of the films upon contact with water.

Protein hydrolysis, polysaccharide blending, and nanofiller reinforcement have further expanded the formulation range of rapeseed protein films. Zhang et al. [189] combined rapeseed protein hydrolysates with chitosan. Moderate hydrolysis improved the compatibility between the rapeseed protein hydrolysates and chitosan under acidic conditions, produced a denser film structure, and increased tensile strength and antimicrobial activity. Osorio-Ruiz et al. [190] incorporated cellulose nanocrystals into a rapeseed protein isolate matrix, improving the mechanical properties and thermal stability of the resulting films. Dissanayake et al. [191,192] incorporated 2,2,6,6-tetramethylpiperidin-1-yl (TEMPO)-oxidized nanocrystalline cellulose and oleic-acid-grafted nanocrystalline cellulose into rapeseed protein matrices. TEMPO-mediated oxidation selectively converts primary hydroxyl groups on the cellulose surface into carboxyl groups. The addition of TEMPO-oxidized nanocrystalline cellulose improved filler dispersion within the rapeseed protein matrix and increased film tensile performance and thermal stability, whereas oleic-acid-grafted nanocrystalline cellulose enhanced film extensibility and moisture barrier properties. Chairez-Jimenez et al. [193] incorporated graphite oxide nanoparticles with different degrees of oxidation into a rapeseed protein matrix. The tensile strength, elastic modulus, and gas-barrier properties of the resulting films varied with the surface chemistry of the graphite oxide. Abookleesh et al. [194] jointly incorporated cellulose nanocrystals, montmorillonite, and hydroxyapatite into a rapeseed protein matrix, producing compression-molded composite films with high mechanical strength and storage stability. Nanofillers can improve the dry-state properties of rapeseed protein films, although filler dispersion, protein–filler interfacial interactions, and performance retention under high-humidity conditions remain dependent on the type and level of filler modification.

Rapeseed protein films and coatings have been evaluated during fruit storage. Jang et al. [195] incorporated grapefruit seed extract into rapeseed protein–gelatin composite films. Packaging strawberries with these films reduced microbial counts and delayed quality deterioration during storage. Formulation-optimized rapeseed protein–gelatin coatings also slowed quality deterioration during fruit storage [196]. Incorporation of montmorillonite and citric acid into rapeseed protein–chitosan composite films improved their mechanical, barrier, and antioxidant properties and delayed water loss and decay in stored blueberries [197]. However, most films and coatings were still produced by batch casting or laboratory-scale coating; data on continuous film production, coating uniformity, migration of active components, food-contact safety, and end-of-life management remain limited.

In addition to solution-cast and compression-molded films, rapeseed proteins have been used in thermoplastic processing and molecularly assembled materials. Manamperi et al. [198] removed protein fractions with high water absorption and poor thermal performance from rapeseed protein isolate and used the refined protein in protein–polyester blended plastics. Materials prepared from the refined protein exhibited higher tensile and flexural strength, toughness, and water resistance, indicating that protein fraction composition affects material performance after thermoplastic processing. However, the system still depended on an external polyester to form a processable continuous phase. Bagnani et al. [199] obtained a cruciferin-enriched protein fraction from rapeseed meal and assembled the protein into amyloid fibrils. Combining the fibrils with polyvinyl alcohol and glycerol produced flexible films with reduced water absorption, greater surface hydrophobicity, and higher elongation at break. This approach used the molecular self-assembly capacity of rapeseed protein, although the continuous film structure and mechanical performance were jointly maintained by the protein fibrils, polyvinyl alcohol, and plasticizer.

Wood adhesives represent another relatively well-studied non-food application of rapeseed protein. Unfolding of the protein chains exposes polar and hydrophobic groups that can interact with wood surfaces through hydrogen bonding, hydrophobic interactions, and other intermolecular forces. Unmodified protein adhesives, however, generally exhibit high viscosity, low wet bond strength, and insufficient water resistance. Li et al. [200] modified rapeseed protein with sodium bisulfite. Cleavage of disulfide bonds altered the protein structure and improved adhesive flow and handling, while low additive levels also increased wet bond strength at some curing temperatures. Wang et al. [201] conjugated rapeseed protein isolate with poly (glycidyl methacrylate), forming a protein–polymer network that increased both dry and wet bond strength. Bandara et al. [202] incorporated graphite oxide into rapeseed protein adhesives, and interactions between the protein and nanomaterial improved bonding strength and water resistance.

Studies of rapeseed protein adhesives in wood-based panels have also examined adhesive formulation and processing conditions. Yang et al. [203] combined rapeseed protein with glyoxal–urea resin and a small amount of isocyanate. When used in plywood, the resulting adhesive provided high bonding strength and low formaldehyde emission. Tene Tayo et al. [204] compared different cross-linkers and reaction times in a rapeseed protein–gelatin–urea system. The sodium nitrite-cross-linked formulation produced relatively high internal bond strength in single-layer particleboards. Adhesive loading, hot-pressing temperature, and pressing time also affected internal bond strength, bending performance, and thickness swelling after water absorption [205]. A small amount of polyethyleneimine improved the mechanical performance of three-layer particleboards by reacting with the rapeseed protein adhesive [206]. Adhesive viscosity and bonding performance changed during storage. Adhesives prepared from protein isolates generally showed better performance and storage stability than systems based directly on rapeseed meal, although panel properties declined with increasing adhesive storage time in both cases [207].

The self-assembly capacity of rapeseed storage proteins has also been used to construct nanoscale delivery carriers. Akbari and Wu [208] induced the formation of cruciferin nanoparticles using calcium ions and evaluated particle size, surface properties, and the potential for encapsulating bioactive compounds. Hong et al. [209] found that amphipathic peptides generated through cruciferin hydrolysis participated in nanoparticle assembly and stabilization. Studies using Caco-2 cells and Caco-2/HT29-MTX co-cultures further evaluated interactions between cruciferin nanoparticles and the intestinal epithelium and mucus layer [210]. Wang et al. [211] constructed self-assembling nanogels from acylated rapeseed protein for the encapsulation of hydrophobic curcumin and improved its dispersion stability in aqueous media.

Rapeseed proteins can also form three-dimensional water-absorbing networks and modify foam properties at gas–liquid interfaces. Shi et al. [212] prepared cross-linked rapeseed protein-based superabsorbent hydrogels capable of absorbing and retaining large amounts of water, supporting their potential use as absorbent materials and controlled-release matrices. Direct evidence for cosmetic applications remains limited. Rivera et al. [213] recovered protein- and peptide-containing active fractions from rapeseed oil-processing co-streams and evaluated their antioxidant and protective effects in skin care applications. However, the tested materials were multicomponent co-stream extracts; the observed activities may not be attributed exclusively to rapeseed protein. Sánchez-Vioque et al. [214] used acylation to modify the surface activity and foam stability of rapeseed protein hydrolysates, while Larré et al. [215] examined the relationships among the degree of hydrolysis, peptide composition, and foaming properties. These studies indicate that rapeseed protein hydrolysates can function in foaming and surface-active systems, although complete formulations and practical evaluations in detergents, personal care products, and industrial foaming agents remain lacking.

The film-forming, adhesive, self-assembling, and water-absorbing properties of rapeseed proteins have been demonstrated in multiple material systems. Films and wood adhesives have been studied relatively systematically, whereas delivery carriers, hydrogels, and surface-active systems have mainly undergone functional evaluation. Future studies may clarify how protein composition and the isolation and modification processes affect performance in specific material applications, reduce dependence on synthetic polymers and high loadings of cross-linkers, and validate water resistance, hygrothermal stability, and long-term performance under continuous processing conditions. Process scale-up should also consider protein recovery, variability in raw material composition, production costs, biodegradation, and life-cycle performance to define the practical application range of each material route.

### 4.3. Bioactive Compounds

Beyond proteins, fractions recovered from rapeseed meal have been evaluated as food ingredients, prebiotic materials, antioxidants, and antimicrobial agents.

Early studies on rapeseed meal polysaccharides focused mainly on antioxidant activity. Zhu and Wu [42] isolated a water-soluble polysaccharide, WPS-1, and an alkali-soluble polysaccharide, APS-2, both of which showed free-radical-scavenging activity. Sun et al. [43] separated the crude polysaccharides into PRM1 and PRM2 and reported radical-scavenging, reducing, and lipid peroxidation-inhibitory activities. Oral administration of a rapeseed meal polysaccharide preparation for 30 days increased glutathione peroxidase, catalase, superoxide dismutase, and total antioxidant capacity in mice and reduced the malondialdehyde level [54]. The polysaccharide preparations retained different amounts of associated protein, and the contribution of the carbohydrate component to the measured antioxidant effects was not resolved.

Prebiotic activity has been examined using purified polysaccharides and pectic oligosaccharides. Wang et al. [55] obtained two protein-bound polysaccharide fractions, RP1 and RP2, with molecular weights of 28.51 and 6.55 kDa, respectively. Both fractions resisted digestion by simulated gastric juice and α-amylase and supported the proliferation and acid production of the tested bifidobacteria and lactobacilli. Banjanac et al. [47] recovered a Ca-bound pectic fraction containing 56.8% galacturonic acid and converted the fraction into short-chain pectic oligosaccharides using Pectinex Ultra Passover. In a simplified in vitro screening assay, the oligosaccharide fraction preferentially supported strains belonging to the family Lactobacillaceae. Confirmation in gastrointestinal models, animals, and humans is still required before these fractions can be considered established prebiotic ingredients.

Food applications of fiber-rich fractions have mainly used rapeseed protein-fiber concentrate rather than purified dietary fiber. Ostrowska et al. [216] compared rapeseed protein-fiber concentrate with commercial soy and pea fibers. The rapeseed concentrate contained more protein than either commercial fiber and had a total dietary fiber content close to that of soy fiber. It also showed water and oil absorption capacities, emulsifying activity, and emulsion stability suitable for use in formulated foods. Axentii et al. [217] replaced 5% to 20% of wheat flour with rapeseed protein-fiber concentrate in pasta. Addition at 5% to 15% increased dough water absorption, stability, and storage modulus. At higher incorporation levels, the continuity of the gluten network deteriorated, cooking loss reached 8.8% to 10.4%, and the firmness of cooked pasta decreased. Because the concentrate contains substantial amounts of both protein and fiber, its hydration and textural effects cannot be assigned to the fiber component alone.

Among the non-protein fractions, phenolic extracts have received the broadest application testing. Thiyam et al. [218] added rapeseed meal extracts to rapeseed oil from which endogenous antioxidants had been removed. At an equivalent total phenolic concentration, the extract inhibited conjugated diene formation to an extent comparable to that of sinapic acid. Vuorela et al. [219] prepared phenolic fractions using aqueous methanol, aqueous ethanol, hot water, and ferulic acid esterase. All fractions reduced the formation of hexanal and conjugated diene hydroperoxides in liposomes and inhibited the oxidation of low-density lipoprotein. Thiyam et al. [220] subsequently examined mixtures of rapeseed phenolics and tocopherols and found that antioxidant interactions depended on the concentrations of both components and on the lipid system. Radical-scavenging assays alone therefore provide limited information on the behavior of rapeseed phenolic extracts in tocopherol-containing oils.

The antioxidant effects of rapeseed meal phenolics have also been examined in meat products, bulk oils, and emulsions. Vuorela et al. [221] added rapeseed phenolic fractions to cooked pork patties and reduced lipid oxidation and protein carbonyl formation during illuminated refrigerated storage. Salminen et al. [222] obtained similar inhibition after adding 0.5 to 0.7 g of rapeseed meal per 100 g of meat. The latter experiment used whole meal, so proteins and other meal components may have contributed to the response. Szydłowska-Czerniak et al. [223] fortified refined rapeseed oil with a phenolic-rich meal extract. The fortified oil had higher total phenolic content and antioxidant capacity than the untreated oil, although both measures declined during storage. Fan et al. [224] reported improved oxidative stability of soybean oil containing 0.014% rapeseed meal extract during storage at 68 °C for 19 days. Increasing the extract concentration did not produce a consistent additional effect. Lee and Lee [225] added hydrolyzed rapeseed press meal extract rich in sinapic acid to fish oil-in-water emulsions at concentrations from 200 to 1000 mg/kg. Lipid hydroperoxide formation decreased with increasing extract concentration during 29 days of refrigerated storage.

Rapeseed meal extracts have also been tested in frying systems and food-packaging materials. Rabiej-Kozioł and Szydłowska-Czerniak [226] added 200 mg/kg of an acid-hydrolyzed and freeze-dried rapeseed meal extract to refined rapeseed oil. The extract reduced oxidative deterioration during heating at 180 °C and during French-fry production, with lower oxidation also detected in the fried product. Tymczewska et al. [227] incorporated water and aqueous methanol extracts of rapeseed meal into gelatin films at concentrations from 4% to 12%. Both extracts increased the radical-scavenging activity and Young’s modulus of the films, and the films were tested against five food-associated bacterial strains. Extract addition also altered film color, opacity, and mechanical behavior. Most food and packaging studies have used crude or partially purified extracts, and differences in phenolic composition, color, flavor, and interactions with the product matrix remain important formulation variables.

Targeted conversion of sinapic acid derivatives has been used to obtain products with greater affinity for lipid phases. Odinot et al. [228] treated rapeseed meal with a feruloyl esterase from *Aspergillus niger*, releasing 6.6 to 7.4 mg of free sinapic acid per gram of meal. The recovered sinapic acid was converted by *Neolentinus lepideus* through non-oxidative decarboxylation, producing 1.3 g/L canolol at a molar yield of 80%. Li and Guo [151] converted sinapine directly into alkyl sinapates through transesterification. Longer-chain alcohols gave lower ester yields but higher product purities. These processes established routes for producing canolol and lipophilic sinapate esters from rapeseed meal, but application data for the purified products in formulated foods remain scarce.

Lan et al. [229] recovered a phytic acid-rich fraction with purity of 45.96% from the detoxification stream generated during rapeseed meal processing. Addition of the fraction to lard reduced the acid and peroxide values and produced measurable scavenging of hydroxyl, superoxide, DPPH, and hydrogen peroxide radicals. More than half of the recovered material consisted of co-extracted compounds, so the independent contribution of phytic acid was not established. GLS applications have focused on enzymatically generated isothiocyanates. Liu et al. [230] converted rapeseed meal-derived GLS using myrosinase obtained from rapeseed sprouts. GLS conversion reached 70%, and 3-butenyl isothiocyanate accounted for approximately 75% of the enzymatic products. The hydrolysate inhibited *Streptococcus agalactiae* at a minimum inhibitory concentration of 64 μg/mL, and β-cyclodextrin encapsulation improved the thermal and storage stability of the isothiocyanates. The antimicrobial evaluation was limited to an in vitro bacterial model, and performance, sensory effects, and non-target hydrolysis products have not been examined in food matrices.

### 4.4. Thermochemical Valorization

Rapeseed meal can be converted through pyrolysis, combustion, gasification, and hydrothermal treatment into liquid products, fuel gases, carbonaceous materials, and soluble low-molecular-weight compounds. The contents of residual oil, protein, structural carbohydrates, and ash vary considerably among these feedstocks. Residual oil can increase the yield and heating value of pyrolysis liquids, whereas protein decomposition generates nitrogen-containing compounds in the bio-oil and gaseous products and retains part of the nitrogen in the char. Feedstock origin and upstream treatments, including oil removal and protein extraction, should therefore be considered when thermochemical conversion data are compared.

Fixed-bed and flash pyrolysis have both been applied to rapeseed press meal. Özçimen and Karaosmanoğlu [231] pyrolyzed cold-pressed rapeseed meal under static conditions and obtained combustible liquid products and carbon-rich solid with relatively high heating value. Uçar and Özkan [232] conducted fixed-bed pyrolysis at 400–900 °C. The bio-oil yield increased up to 500 °C and then decreased slightly. The bio-oil produced at 500 °C had a higher heating value of 33.17 MJ/kg and contained oleic acid, fatty acid amides, indole, phenolic compounds, and aromatic hydrocarbons, while CO_2_, CO, hydrocarbons, and H_2_S were detected in the gas fraction. Smets et al. [233] applied flash pyrolysis at 350–550 °C. The oil-phase yield reached 42.1% at 550 °C, with a water content of 6.7% and a higher heating value of 32.8 MJ/kg. Residual triglycerides and related compounds from the feedstock represented the main components of the oil phase, indicating that rapid heating promoted lipid volatilization while extensive triglyceride cracking remained limited.

The pyrolysis behavior of de-oiled rapeseed meal is influenced by its protein and ash contents. Azargohar et al. [234] conducted slow pyrolysis at 300–700 °C. The bio-oil yield ranged from 10% to 24% and reached its maximum at 500 °C, while the biochar yield decreased and the gas yield increased continuously with temperature. The resulting chars contained 6–9% nitrogen, and the char prepared at 400 °C had a higher heating value of 29.8 MJ/kg. Sun et al. [154] examined the effects of heating rate and residence time. Under intermediate pyrolysis conditions, the bio-oil yield increased from 43.2% at 350 °C to 58.9% at 650 °C, whereas the gas yield during slow pyrolysis increased from 4.0% to 14.5%. Most organic components in rapeseed meal had decomposed at approximately 500 °C, and the bio-oil contained substantial amounts of amides and pyrrole derivatives. Residues remaining after protein extraction can also be subjected to pyrolysis. Gallorini et al. [153] modified the induction–pyrolysis reactor configuration and operating conditions to obtain bio-oil and gas yields of 30.5% and 30.7%, respectively, or to increase the char yield to 53%. Pyrroles, pyrazine derivatives, and furfuryl alcohol were detected in the bio-oil, reflecting the combined decomposition of residual nitrogen-containing compounds and carbohydrates.

The oxygen, nitrogen, and sulfur present in rapeseed cake bio-oil restrict its direct use as a fuel. Catalytic vapor treatment and hydrorefining have therefore been applied to improve the liquid composition. Giannakopoulou et al. [235] placed an H-ZSM-5 or H-Beta catalytic bed downstream of the pyrolysis reactor. H-ZSM-5 promoted hydrocarbon formation and showed greater stability, whereas H-Beta exhibited stronger deoxygenation activity. Smets et al. [236] compared Na_2_CO_3_, γ-Al_2_O_3_, and HZSM-5. All three catalysts decreased the liquid yield and increased the formation of water and non-condensable gases. HZSM-5 provided a relatively high degree of deoxygenation, while the oil phase obtained with Na_2_CO_3_ had a relatively high heating value. Under catalytic conditions, fatty acids were further converted into nitriles, aromatic compounds, and aliphatic hydrocarbons.

Catalyst selection also affects the conversion of nitrogen-containing compounds and catalyst deactivation. Pstrowska et al. [237] hydrorefined rapeseed cake pyrolysis oil over a NiMo/Al_2_O_3_ catalyst. One-step treatment of the original bio-oil did not provide sufficient upgrading. After removal of the low-boiling fraction and reduction of the liquid hourly space velocity to 0.5 h^−1^, the desulfurization, denitrogenation, and deoxygenation efficiencies reached 71.4%, 29.0%, and 78.8%, respectively. The resulting product was still unsuitable for direct use in internal combustion engines. A co-refining mixture containing 20% bio-oil and petroleum fraction produced fuel components with improved properties. David and Kopač [238] treated pyrolysis vapors with Fe_2_O_3_/ZSM-5. The catalyst decreased the bio-oil yield, increased gas and water formation, and promoted deoxygenation and the formation of aromatic and phenolic compounds. Jerzak et al. [239] repeatedly used ZSM-5 and Zeolite Y during the treatment of post-extraction rapeseed meal. After ten cycles, the amount of coke deposited on Zeolite Y was approximately five times that deposited on ZSM-5, while ZSM-5 produced bio-oil with lower oxygen content. Deoxygenation, nitrogen-containing products, liquid yield, and catalyst coking should therefore be considered together when catalytic upgrading processes are evaluated.

Rapeseed meal can also be combusted directly as solid fuel. Eriksson et al. [240] determined the fuel properties of rapeseed meal obtained from several industrial sources. Rapeseed meal contained approximately 74% volatile matter, 6.2–11.8% moisture, and about 7.4% ash. Its ash, nitrogen, sulfur, phosphorus, and potassium contents were higher than those of most woody fuels. Relatively high NO and SO_2_ emissions were observed during combustion, and pulverized combustion also generated a large amount of fine particulate matter. Boström et al. [241] further investigated ash transformations during the bubbling fluidized-bed combustion of rapeseed meal and bark. Phosphates dominated the rapeseed meal ash, whose chemical composition differed from that of silicate-rich woody biomass. Severe bed agglomeration was not observed under the tested conditions, although the transfer of phosphorus, potassium, and sulfur affected fly ash composition and flue gas cleaning requirements.

Chars produced from rapeseed meal can undergo further conversion with CO_2_ or steam. Nowicki et al. [242] measured CO_2_ gasification kinetics at 800–900 °C. The random pore model provided an adequate description of char conversion, with an apparent activation energy of 222.1 kJ/mol and a reaction order of 0.57 with respect to CO_2_. Steam gasification at 750–850 °C gave an apparent activation energy of 166 kJ/mol and a steam reaction order of 0.5 [243]. Both studies were conducted using thermogravimetric analysis and laboratory-prepared chars. The reported results provide kinetic parameters but do not establish syngas balances, tar formation, or mineral-element transfer under continuous gasification conditions.

Chemical looping gasification has been evaluated using Ca–Fe oxygen carriers. Niu and Shen [244] found that increasing the oxygen-carrier-to-rapeseed-cake ratio enhanced carbon conversion. NH_3_ and HCN were the principal gaseous nitrogen products. Niu et al. [245] applied torrefaction at 200–300 °C before chemical looping gasification. Torrefaction decreased the H/C and O/C ratios, increased energy density, and improved gasification reactivity. Some thermally unstable nitrogen groups were released during pretreatment, while the subsequent formation of NH_3_ and HCN decreased only slightly.

Subcritical water processing allows simultaneous decomposition of proteins, structural carbohydrates, and residual lipids. Pińkowska et al. [246] used a Doehlert experimental design to determine suitable hydrothermolysis conditions for rapeseed meal. Treatment at approximately 205 °C for 51 min produced an amino acid yield corresponding to 32.8% of the protein content, whereas greater xylose and glucose release required a higher temperature and shorter residence time. Hydrothermolysis of rapeseed cake also generated amino acids and free fatty acids [247]. Increasing the reaction severity promoted amino acid deamination and decarboxylation, Maillard reactions, and the isomerization of unsaturated fatty acids. Remón et al. [248] applied acetic-acid-catalyzed microwave hydrothermal treatment. Processing at 186 °C for 3 min in 1 mol/L acetic acid produced a lignin-rich solid with a purity of approximately 85% and an aqueous fraction rich in oligosaccharides. These processes were designed for component decomposition and fractionation rather than the production of a fuel-type biocrude.

Solid products obtained from pyrolysis have been evaluated as adsorbents and soil amendments. Rambabu et al. [249] activated de-oiled canola meal char using steam, CO_2_, NH_3_, or KOH. The KOH-activated carbon reached a maximum ammonium adsorption capacity of 148 mg/g. David [250] prepared H_2_SO_4_- and KOH-activated chars from rapeseed meal. The acid-activated carbon adsorbed approximately 166 mg/g toluene and 152 mg/g acetone. Kwoczynski et al. [251] combined biochar produced from extracted rapeseed meal with digestate in a lettuce pot experiment. Biochar increased soil cation-exchange capacity and available potassium, and the biochar treatment produced the highest plant biomass among the treatments examined. Field-scale studies are still needed to determine nutrient-release patterns, contaminant risks, and carbon stability under long-term soil conditions.

Native nitrogen and sulfur can also be incorporated into the carbon framework during carbonization. Zhang et al. [252] prepared N- and S-doped porous carbon by ZnCl_2_ activation followed by carbonization at 900 °C. The material showed oxygen-reduction activity and was tested as a cathode catalyst in a microbial fuel cell. Rao et al. [253] sulfonated partially carbonized de-oiled rapeseed meal to produce a solid acid catalyst for the esterification of oleic acid and high-free-fatty-acid rapeseed oil. Catalyst activity declined during repeated use and could be restored by resulfonation. Zhang et al. [254] prepared sulfonated porous carbon through KOH activation and grafting with 1,3-propanesultone. The catalyst converted 95.6% of oleic acid after 4 h at 70 °C, and conversion decreased from approximately 95% to 90% after five cycles.

Most studies on thermochemical valorization of rapeseed meal have been conducted in batch reactors or at laboratory scale. Differences in residual oil, protein, and ash contents have not yet been resolved through standardized mass and energy balances. For fuel-oriented pathways, nitrogenous products formed during protein decomposition increase the requirements for product upgrading and emission control. Nitrogen retained in the solid phase may instead support the production of heteroatom-doped carbon materials. Further work should address continuous operation, catalyst regeneration, tar and emission control, separation of hydrothermal products, activation-agent recovery, washing-water demand, and the yield of the final functional carbon materials.

### 4.5. Regulatory Guidelines and Commercial Products

Rapeseed meal is recognized as a feed material under the EU regulatory framework. The EU Catalogue of Feed Materials lists rapeseed meal as a feed material and requires declaration of its crude protein content [255]. For rapeseed meal feed produced at integrated oil processing and refining facilities, the maximum permitted contents of spent bleaching earth and filter aids, crude lecithins, and soap stocks are 1%, 1.3%, and 2%, respectively [255]. Regulation (EC) No 767/2009 governs the placing on the market and use of feed, while Regulation (EC) No 183/2005 establishes hygiene requirements for feed production and requires HACCP-based procedures for activities other than primary production [256,257]. Directive 2002/32/EC sets maximum permitted levels for lead, cadmium, arsenic, mycotoxins, and other undesirable substances in animal feed [258]. No specific maximum level for GLS in rapeseed meal is given in the current EU Catalogue of Feed Materials or Directive 2002/32/EC [255,258].

Rapeseed-derived ingredients intended for human consumption are regulated under the general EU legislation on food safety and food hygiene. Products that meet the definition of a novel food require authorization under Regulation (EU) 2015/2283 and inclusion in the Union list of novel foods [259,260,261,262]. Rapeseed protein was authorized as a novel food ingredient in 2014 [263]. The authorized specifications require at least 90% total protein and 85% soluble protein, with maximum contents of 2% fat, 0.5% fiber, 1 mmol/L total GLS, 1.5% phytic acid, and 0.5 mg/kg lead [263].

The EU has also authorized rapeseed powders containing substantial amounts of non-protein components. Partially defatted rapeseed powder was authorized as a novel food in 2021, and its specifications were revised in 2025 to 28–43% protein, 10–22% lipid, and 33–46% total dietary fiber, with total GLS below 0.3 mmol/kg (≤120 mg/kg) and phytic acid below 1.5% [264,265]. Defatted rapeseed powder authorized in 2026 is produced by aqueous extraction followed by treatment with ethanol and ethyl acetate [266]. The product contains 28–45% protein and 37–70% total dietary fiber, while fat, total GLS, and phytic acid are limited to 2%, 0.1 mmol/kg (≤40 mg/kg), and 2%, respectively; residual ethanol and ethyl acetate must each remain below 200 mg/kg [266]. The maximum levels of lead, total arsenic, cadmium, mercury, and aluminum are 0.2, 0.1, 0.1, 0.02, and 35 mg/kg, respectively [266]. Authorized applications include bakery products, breakfast cereals, cereal bars, meat alternatives, and meat products, with maximum use levels of 20 g/100 g for most cereal and bakery products and 15 g/100 g for meat alternatives and meat products [266].

Commercial feed and food ingredients derived from rapeseed meal and its fractions are already available. Bunge markets rapeseed meal as a feed material [267]. In 2022, dsm-firmenich launched Vertis™ CanolaPRO^®^, a rapeseed protein isolate produced from a co-product of rapeseed oil processing at a newly constructed production facility in Dieppe, France [268]. Vertis™ CanolaPRO^®^ contains more than 90% protein [269] and has a protein digestibility-corrected amino acid score (PDCAAS) of 1 [269,270]. Applications include plant-based meat and dairy alternatives, protein beverages, protein bars, cereals, and pasta products [270]. Apetit developed BlackGrain^®^ rapeseed powder from a side stream of rapeseed oil production. The commercial ingredient contains approximately 34% plant protein, 39% dietary fiber, and 21% fat [271]. BlackGrain^®^ is also used to produce a textured vegetable protein containing 20% BlackGrain^®^ rapeseed powder and 80% pea protein isolate. The product contains approximately 70% protein, 6% fiber, and 10% fat and is mainly intended for meat-alternative products [271].

## 5. Application and Process Integration of Rapeseed Meal Fractions

**Figure 3:**
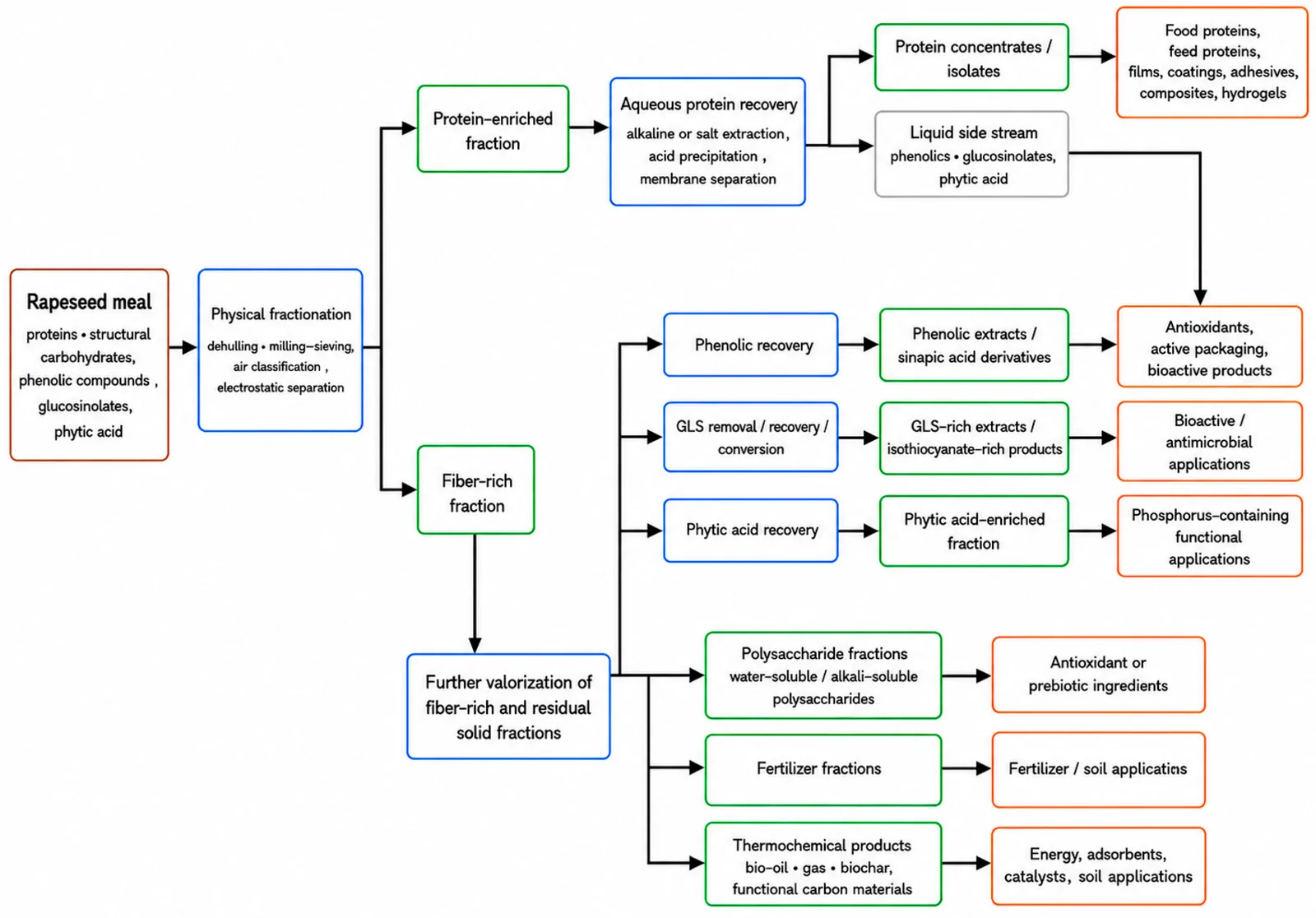
Integrated valorization scheme of rapeseed meal showing sequential fraction recovery and potential applications.

To provide an integrated perspective on rapeseed meal valorization, the sequential recovery of its major fractions and their potential applications is summarized in Figure 3.

This section compares the strength of the available evidence across utilization routes and examines how fractionation can be integrated with downstream use. Table 2 summarizes the applications that have been evaluated for rapeseed meal and its fractionated or processed products, together with the main questions that remain unresolved. The feasibility of a fractionation route depends not only on component recovery, but also on whether the resulting material has defined product characteristics, delivers reproducible performance in the intended application, and generates sufficient value to justify the additional processing requirements.

The extent of application validation differs considerably among utilization routes. Feed, fertilizer, food protein, protein films, and wood adhesives have been evaluated through animal feeding trials, pot or field experiments, food processing studies, or panel performance tests [56,155,156,157,158,159,160,161,162,163,164,165,166,167,168,169,170,171,172,173,174,175,176,177,178,179,180,181,182,183,184,185,186,187,188,189,190,191,192,193,194,195,196,197,198,199,200,201,202,203,204,205,206,207]. These studies provide direct evidence of whether compositional changes introduced by processing or fractionation improve animal performance, crop growth, food quality, or material properties. Phenolic extracts have also been tested in edible oils, frying systems, and packaging films, whereas application data for purified sinapic acid derivatives and canolol remain limited [151,220,221,222,223,224,225,226,227,228]. Based on the available application evidence, the use of rapeseed meal phenolic extracts as natural antioxidants in lipid-containing food systems, particularly edible oils and frying systems, appears to be the most immediately applicable route. By comparison, polysaccharides, GLS conversion products, phytate-rich fractions, protein delivery systems, and hydrogels have mainly been assessed through compositional analysis, in vitro assays, stability tests, or laboratory-scale functional evaluation [42,43,54,55,208,209,210,211,212,213,214,215,229,230]. Routes supported by application evidence therefore require greater attention to product consistency and scale-up, whereas less developed routes first require clearer product definitions and suitable target applications.

Product specifications should be established according to the intended use of each fraction. Protein content or purity alone does not predict food or material performance. The cruciferin/napin ratio, aggregation state, solubility, and residual contents of phenolic compounds, phytate, and fiber can all affect functionality [56,162,163,164,165,166,167,168,169,170,171,172,173,174,175,176,177,178,179,180,181,182,183,184,185,186,187,188,189,190,191,192,193,194,195,196,197,198,199,200,201,202,203,204,205,206,207,208,209,210,211,212,213,214,215]. Polysaccharide and phytate-rich fractions may retain bound proteins, phenolic compounds, minerals, and other co-extracted constituents, which can contribute to the measured antioxidant or prebiotic effects [42,43,54,55,229]. The utilization of GLS also depends on the relative formation of isothiocyanates, nitriles, and oxazolidine-2-thiones, because product composition determines both activity and safety [230]. For thermochemical conversion, relevant feedstock characteristics include residual oil, protein, ash, nitrogen, and sulfur contents, all of which influence the yields and compositions of bio-oil, gas, and carbonaceous products [153,154,231,232,233,234,235,236,237,238,239,240,241,242,243,244,245,246,247,248,249,250,251,252,253,254]. Fractionation processes should therefore be evaluated using composition and performance criteria that are relevant to the final application, rather than extraction yield, total protein, total phenolic content, or GLS degradation alone.

The required degree of separation and purification also depends on the downstream application. Food proteins require reductions in bitterness, astringency, off-flavor, and antinutritional factors while retaining solubility, emulsifying capacity, and structuring performance. Extensive purification may increase water and chemical consumption and may also alter protein composition in ways that reduce processing functionality. Mixed phenolic extracts may be suitable for certain oil and packaging applications, whereas purified compounds are more appropriate when composition and dosage must be tightly controlled. Feed, fertilizer, and thermochemical routes generally tolerate greater compositional complexity and can utilize enriched fractions or solid residues that are unsuitable for food or high-specification material applications. The extent of fractionation should therefore be determined by the requirements of the intended product, since high purity is not necessary for every utilization route.

Recovery of one component also affects the distribution and quality of other components. During aqueous protein extraction, phenolic compounds, GLS, and phytate may enter the liquid phase or remain associated with proteins, whereas fiber and most structural polysaccharides are retained in the solid residue. Acid precipitation, membrane separation, adsorption, and ion exchange can further redistribute target and non-target compounds. Increasing the recovery of one product may reduce the purity or yield of another product and may increase the treatment burden associated with residual liquid streams. Process design should therefore consider the main product, co-products, and remaining solid and liquid streams together, with processing order selected according to component stability, interactions, and downstream use.

From a process-integration perspective, physical fractionation is compatible with subsequent selective wet extraction: it can first generate protein-enriched and fiber-enriched fractions without introducing substantial liquid side streams, after which proteins or other components can be further recovered according to the target product. However, physical fractionation primarily achieves enrichment rather than high-purity separation, and its performance is constrained by incomplete tissue liberation and overlap in the physical properties of protein- and fiber-rich particles; therefore, wet separation is generally still required when high-purity products are targeted.

For routes targeting food proteins, mild treatments that preserve protein functionality should be prioritized, and protein recovery should preferably precede strongly alkaline, high-temperature, or hydrothermal treatment. Phenolic compounds, GLS, and phytic acid can either be recovered directly as target products or further separated from liquid streams generated during protein extraction. However, increasing the purity of these fractions generally requires additional precipitation, membrane separation, adsorption, or solvent-based steps, thereby increasing water, chemical, and energy demand and the burden of liquid-stream treatment. Thus, not every technically recoverable fraction necessarily justifies recovery as a separate product; its potential value should be considered against the additional separation and purification requirements.

The quality requirements of different applications also determine the appropriate degree of fractionation. Food applications generally require relatively high purity, acceptable sensory quality, and retention of protein functionality; however, increasing protein purity may involve trade-offs with solubility, emulsifying properties, and structural integrity, and excessive extraction or purification can impair food processing functionality. Material applications can tolerate a certain degree of structural modification and compositional complexity provided that the required film-forming, adhesive, or mechanical performance is achieved; however, lower feedstock purity does not necessarily simplify downstream processing, because some protein-based materials still require cross-linking, blending, or reinforcement to achieve sufficient water resistance, mechanical strength, and long-term stability. Feed applications are more suitable for low-fiber, protein-enriched fractions in which antinutritional factors are controlled, whereas high-fiber fractions or residues subjected to intensive chemical treatment may be less appropriate for feed.

Thermochemical conversion is particularly suitable as a terminal step in a cascading valorization scheme. For complex solid residues remaining after the recovery of proteins, phenolic compounds, or other higher-value fractions, the technical and economic justification for further fine separation may diminish, whereas pyrolysis, gasification, or carbonization can accommodate more compositionally heterogeneous feedstocks and further convert residual organic matter into fuels, chemicals, biochar, or functional carbon materials. However, thermochemical conversion is also constrained by residual feedstock composition. Residual protein and relatively high nitrogen and sulfur contents may increase the formation of nitrogen- and sulfur-containing products and the need for downstream upgrading and emission control, while redistribution of ash and minerals during upstream fractionation may also alter thermochemical behavior and final product properties. Therefore, compared with direct thermochemical conversion of whole rapeseed meal, positioning it after the recovery of higher-value fractions may improve the overall utilization of the feedstock.

Selection of the final route should consider product performance, overall component utilization, and processing demand. Therefore, there is no single fractionation sequence that is suitable for all target products. A reasonable principle is to preserve and recover fractions that are sensitive to processing conditions and offer higher application value first, and then direct the remaining streams to material, feed, soil, or thermochemical applications according to their composition and quality. However, excessive fractionation increases the requirements for extraction, purification, and drying, which may reduce the overall advantages of cascading valorization. Future studies should establish mass balances for the main product, co-products, and residual streams, while also evaluating water, chemical, and energy consumption and the stability of continuous operation. The fractionation route will have a practical basis for scale-up only when the resulting products show reproducible advantages in their intended applications, and the remaining components can be utilized effectively.

## 6. Conclusions and Future Perspectives

Rapeseed meal is produced in large quantities and is a relatively inexpensive raw material containing proteins, carbohydrates, and other valuable components, making it suitable for further development and utilization. Different oil production processes generate rapeseed meals with distinct compositions and structural states, which can subsequently affect component accessibility and recovery. Although considerable research has been conducted on the separation and utilization of rapeseed meal components, many studies still focus on the recovery of a single component or the development of a single product. Accordingly, this review provides an integrated overview of how rapeseed meal is produced, how the major components are fractionated and recovered, and how the derived products are currently utilized. Based on the evidence reviewed here, we consider carbohydrate valorization to warrant particular research priority. Carbohydrates comprise approximately 35% of rapeseed meal dry matter [9], yet research on their selective recovery, detailed characterization, and high-value utilization remains limited. Their conversion into polysaccharides, oligosaccharides, and other products with defined composition and functionality therefore represents a particularly promising direction for further valorization. Major technical limitations remain, including high water and chemical demand, losses of functionality during intensive extraction, incomplete separation of coexisting components, and limited application-level validation.

Future research may give particular attention to improving the selectivity and efficiency of carbohydrate recovery, clarifying the relationships between carbohydrate composition, molecular characteristics, and functionality, and extending current studies beyond mainly in vitro activity assays to digestion, gut microbial utilization, and validation in real food products. Enzymatic and other mild treatments also deserve further investigation for the targeted release, degradation, or modification of rapeseed meal carbohydrates. For proteins, where a larger evidence base already exists, further work should address the effects of extraction and purification on functionality and sensory quality and strengthen validation in actual food and material systems. For phenolic and other bioactive compounds, composition, principal active constituents, and appropriate conditions of use should be better defined; for antinutritional factors, efficient removal or conversion should be achieved while minimizing adverse effects on other valuable fractions. Future fractionation schemes should also consider compatibility between sequential recovery steps and further utilization of residual fractions to improve overall rapeseed meal utilization. Where appropriate, continuous and pilot-scale validation will also be needed to assess practical feasibility.

## Figures and Tables

**Table 1:** Comparison of fractionation, recovery, and process integration strategies for rapeseed meal valorization.

Strategy	Main Components and Fractions	Yield and Recovery	Purity, Selectivity and Functionality	Water, Solvent, and Energy Demand	Scalability and Downstream Compatibility	Side Streams and Process Considerations	References
Milling–sieving	Protein-enriched and fiber-enriched fractions	Moderate/high; the target fine fraction commonly represents about 1/4–1/2 of the feed	Moderate selectivity; increased protein concentration and nutritional value	No process water or solvent; energy mainly for milling and sieving	High; simple equipment, suitable for direct use or further wet refining	No liquid side stream; enrichment is limited by tissue liberation and particle-size overlap	[8,23,30,133]
Milling-air classification	Protein-enriched and fiber-enriched fractions	High; the protein-enriched fraction commonly represents about 1/3–1/2 of the feed	Moderate selectivity; increased protein-to-fiber ratio	No process water or solvent; energy mainly for milling, rotor operation, and air circulation	High; suitable for continuous processing and pre-enrichment	High target-fraction recovery; fine hull particles can reduce protein enrichment	[8,32,33,34]
Milling–electrostatic separation	Protein-enriched and fiber-enriched fractions	Moderate; the target fraction commonly represents about 1/4–2/5 of the feed	Relatively high selectivity; protein solubility can increase with enrichment	No process water or solvent; energy mainly for fine/ultrafine milling and electrostatic separation	Moderate; suitable for combination with air classification	Greater enrichment; sensitive to moisture, residual oil, and particle surface properties	[19,29,34,36]
Water/hot-water extraction	Water-soluble polysaccharides, some proteins, and low-molecular-weight compounds	Moderate; mainly releases readily soluble components	Low/moderate selectivity; relatively good structural preservation	High water demand; energy mainly for heating, stirring, and subsequent concentration/drying	High; liquid streams can be coupled with membrane/adsorption processes and solids can be further valorized	Mild conditions, but dilute extracts increase downstream water removal requirements	[40,42,47]
Acid extraction	Pectin and other acid-soluble polysaccharides	Moderate; varies with acidity, temperature, and extraction time	Moderate selectivity; promotes pectin release, while severe conditions can reduce molecular weight	Requires water and acid; energy mainly for heating, separation, and drying	Moderate/high; based on mature unit operations	Effective for cell-wall polysaccharide release; acid consumption and neutralization salts increase downstream burden	[41,42,47]
Alkaline extraction	Proteins, alkali-soluble polysaccharides, and other bound components	High; strong release of target components	Moderate selectivity; extensive extraction, while high pH can affect color, structure, and functionality	High water and alkali demand; energy for mixing, pH adjustment, separation, concentration, and drying	High; pilot-scale validation available and readily coupled with precipitation and membrane separation	High recovery; co-extraction, chemical consumption, and product quality require balancing	[63,86,87,88,90,91,94]
Salt extraction	Proteins	Moderate/high	Relatively high selectivity; napin/cruciferin distribution can be adjusted while maintaining good solubility	Requires water and salt; energy mainly for mixing and UF/DF desalting	Moderate/high; highly compatible with membrane separation	Mild processing conditions; salt use and desalting remain major process burdens	[63,83,99]
Enzyme-assisted extraction	Proteins, polysaccharides, pectin, and phytate-associated components	Moderate/high; generally higher than aqueous extraction without enzyme pretreatment	Relatively high selectivity; favors mild release and preservation of functionality	Requires water; energy mainly for temperature control, stirring, and reaction time	Moderate; compatible with membrane processing, fermentation, and cascade utilization	High selectivity; enzyme cost and residence time influence process economics	[46,47,65,66,67,68,71]
Ultrasound-, PEF-, or PLE-assisted extraction	Proteins, phenolics, and other extractable components	Moderate; mainly used to increase baseline extraction or shorten processing time	Moderate selectivity; enhances mass transfer, with functional changes depending on treatment intensity	An extraction medium is still required; energy input arises from ultrasound, electric fields, pressure, or heating	Moderate; most rapeseed studies remain at laboratory scale	Strong mass-transfer enhancement; equipment requirements and additional energy input determine the overall benefit	[70,71,72,73]
DES/NADES extraction	Proteins, phenolics, GLS, phytate, and other target compounds	Moderate/high	Relatively high selectivity; favorable retention of color, structure, and some functional properties	Requires DES/NADES and water; energy mainly for heating, mixing of viscous systems, and solvent recovery	Moderate; continuous operation and solvent recycling require further optimization	Good product quality and selectivity; viscosity and solvent recovery are key engineering considerations	[76,77,78,79]
Isoelectric/fractionated precipitation	Proteins	Moderate; fractionated or repeated precipitation can increase overall recovery	Moderate/high selectivity; pH affects protein composition, solubility, and emulsifying properties	Requires water and acid/base; energy mainly for mixing, centrifugation, and drying	High; pilot-scale validation available and readily coupled with UF/DF	Simple bulk recovery; acid-soluble proteins remain in the supernatant	[86,87,88,90,91,92,94]
MF/UF/DF membrane separation	Partitioning of proteins from phenolics, GLS, sugars, salts, and other low-molecular-weight compounds	High; can further increase overall protein recovery from liquid streams	Relatively high selectivity; generally preserves protein solubility and interfacial functionality	UF has low solvent demand, whereas DF can require substantial water; energy mainly for pumping and transmembrane pressure	High; continuous scale-up is well established and highly compatible with extraction and precipitation	Combines concentration and purification; membrane fouling and DF water demand are major operating constraints	[92,95,96,97,98,99]
Adsorption, ion exchange, and chromatography	Proteins, phenolics, phytate, and other target compounds	Moderate; primarily used for highly selective recovery	High/very high selectivity; capable of producing high-purity products	Requires buffers, salts, and/or eluents; energy mainly for pumping, elution, and solvent recovery	Moderate; best suited to downstream purification of higher-value products	High purity and selectivity; additional unit operations, reagents, and regeneration increase process complexity	[100,101,102,105,112,114,115,117]
Sequential multi-component cascade recovery	Proteins, phenolics, polysaccharides, and other co-products	High overall recovery across multiple product streams	Individual steps target different components and can generate multiple functional product streams	Resource demand depends on the combined unit operations and internal stream recycling	Moderate; performance depends strongly on compatibility between consecutive operations	Improves whole-meal utilization; processing order determines the properties of downstream feed streams	[111,113,149,150,151,152]
Fractionated recovery from the same extract	Different protein fractions, phenolics, phytate, and other soluble components	High overall recovery	Relatively high selectivity; precipitation, membrane separation, and adsorption can be applied sequentially	Resource demand arises from repeated pH adjustment, membrane processing, and elution	Moderate/high; individual unit operations are mature	Improves utilization of a single extract; process complexity increases with the number of separation stages	[92,95,101,113]
Valorization of liquid side streams	Phenolics, phytate, and other low-molecular-weight compounds	Moderate/high; depends on target compound concentration in the side stream	High selectivity; enables recovery of high-value low-molecular-weight products	Main consumables are eluents and regenerants; energy mainly for adsorption, concentration, and solvent recovery	Moderate; continuous and semi-continuous approaches have been investigated	Increases the value of liquid side streams; economics depend on target concentration and purification requirements	[105,112,113,114,115,117]
Further conversion of post-extraction solid residues	Fiber, residual proteins, and carbohydrates	Depends on residue conversion and downstream product yield	Low purity requirement; suitable for fermentation or thermochemical conversion	Further intensive wet refining is generally unnecessary; energy demand depends on the downstream route	Moderate/high; compatible with multiple downstream conversion routes, depending on residue composition and upstream processing history	Suitable for complex, low-purity residues; upstream processing affects subsequent conversion performance	[111,133,149,150,151,152,153,154]

**Table 2:** Rapeseed meal feedstocks or fractionated/processed materials, validated applications, and key research needs.

Utilization route	Feedstock or Fractionated/Processed material	Validated Applications/Evaluations	Key Research Needs
Feed	1. Low-fiber, protein-enriched fractions 2. High-fiber fractions	1. Nutrient composition and digestibility 2. Animal feeding, nutritional value, and productive performance	1. Determine whether the compositional advantages of modern dry-fractionated fractions consistently translate into improved animal performance 2. Define appropriate utilization pathways for the protein-enriched and high-fiber fractions 3. Assess whether the benefits of fractionation justify the processing costs
Fertilizer and soil amendment	1. Fermented rapeseed meal and free-amino-acid- and short-peptide-rich fertilizers derived from it 2. Rapeseed meal applied directly to soil 3. Biochar produced from rapeseed meal or residual solid fractions	1. Composition, nutrient release, and detoxification of fermented products 2. Pot and field evaluation of crop growth, yield, and quality 3. Assessment of soil nutrients, physicochemical properties, enzyme activities, and microbial responses 4. Suppression of plant-parasitic nematodes and assessment of phytotoxicity 5. Evaluation of rapeseed meal-derived biochar for soil application, including effects on soil nutrient availability, cation-exchange capacity, and plant growth	1. Relate fermentation extent and the contents of free amino acids, short peptides, inorganic nitrogen, and residual GLS hydrolysis products 2. Define application rate, timing, and the interval before sowing according to nutrient release and crop demand 3. Balance nematode suppression with seed germination, seedling growth, and crop safety 4. Evaluate fertilizer effectiveness, nutrient-use efficiency, soil quality, and economics across crop types, soils, and repeated applications 5. Assess long-term nutrient release, contaminant risks, and carbon stability of biochar used in soil
Food proteins	1. Protein concentrates and isolates 2. Salt-extracted, alkali-extracted, and ultrafiltered proteins 3. Fermented or hydrolyzed proteins	1. Emulsification and lipid structuring 2. Bakery and texturized foods 3. Sensory, digestibility, and nutritional assessment	1. Increase protein recovery and reduce resource use while retaining sensory and functional quality 2. Reduce bitterness, astringency, and off-flavors without impairing solubility, emulsification, or texturization 3. Establish relationships between protein composition, co-extracted compounds, and processing performance in different foods 4. Validate thermal-processing and long-term storage stability in complete food matrices
Protein materials	1. Protein concentrates and isolates 2. Protein hydrolysates and chemically modified proteins 3. Cruciferin-rich and other selectively enriched protein fractions	1. Films, coatings, and packaging applications 2. Wood adhesives and panels 3. Thermoplastic composites 4. Functional evaluation of delivery carriers and hydrogels	1. Clarify how upstream processing history, protein-isolation route, and protein composition affect material performance 2. Improve the mechanical, adhesive, and barrier properties of films and adhesives under water contact and high humidity 3. Maintain material performance while reducing dependence on synthetic polymers, high cross-linker loadings, and nanofillers 4. Extend validation to scale-up processing, long-term use, and application-specific safety and efficacy
Polysaccharides	1. Water-soluble and alkali-soluble polysaccharides 2. Protein-bound polysaccharides	1. In vitro antioxidant activity 2. In vivo antioxidant assessment in mice 3. Stability during simulated gastrointestinal digestion 4. Growth and acidification of selected probiotic strains	1. Clarify the contributions of bound protein and other coexisting constituents to the measured activities 2. Relate polysaccharide composition, molecular weight, and branching features to antioxidant or prebiotic effects 3. Validate the selective fermentation, microbiota changes, and metabolite production of isolated polysaccharides in complex gut microbial communities
Phenolic products	1. Crude and enriched phenolic extracts from rapeseed meal 2. Enriched or purified sinapic acid and sinapine fractions	1. Antioxidant evaluation in model lipid systems 2. Antioxidant validation during edible-oil storage and frying 3. Antioxidant, antimicrobial, and material-performance evaluation of active packaging films	1. Establish product specifications based on individual phenolic composition and purity, and identify the principal active constituents 2. Determine effective use concentrations in target foods and evaluate antioxidant efficacy, color, bitterness, astringency, and matrix interactions 3. Validate the practical performance and stability of high-purity sinapic acid, sinapine, canolol, and alkyl sinapates 4. Verify whether recovery yield, product purity, and throughput remain stable during pilot-scale or continuous operation, and assess solvent recovery, repeated use of adsorption resins, and effects on subsequent fraction utilization
GLS recovery and conversion	1. GLS extracts or enriched fractions from rapeseed meal	1. Enzymatic conversion of GLS into isothiocyanates 2. In vitro antimicrobial evaluation of isothiocyanate-rich hydrolysates 3. Thermal and storage stability after encapsulation	1. Increase selectivity toward target isothiocyanates and quantify residual GLS, nitriles, oxazolidine-2-thiones, and other non-target hydrolysis products 2. Validate antimicrobial spectrum, effective concentration, stability, and safety in the intended application system 3. Clarify how variation in the GLS profile of rapeseed meal affects hydrolysate composition and antimicrobial performance 4. Evaluate overall product yield, processing cost, and use of remaining fractions across GLS extraction, enzymatic conversion, and stabilization
Phytic acid recovery	1. Phytic acid-enriched fractions from rapeseed meal extracts or processing streams	1. Isolation and enrichment of phytic acid 2. Preliminary antioxidant evaluation in one lipid system	1. Increase the purity of phytic acid-enriched fractions and identify co-extracted proteins, phenolics, inorganic salts, and other constituents 2. Confirm the practical function of compositionally defined phytic acid products and use the results to identify suitable applications 3. Integrate phytic acid recovery with protein and phenolic fractionation and evaluate component recovery, reagent demand, and overall process feasibility
Thermochemical production of fuels and chemicals	1. De-oiled rapeseed meal 2. Solid residues remaining after protein extraction	1. Yield, composition, and fuel properties of pyrolysis products 2. Combustion and co-gasification 3. Hydrothermal production of amino acids, sugars, and lignin-rich fractions 4. Catalytic upgrading of pyrolysis products and catalyst reuse	1. Clarify how upstream oil processing and component extraction, together with residual oil, protein, ash, nitrogen, and sulfur contents, affect product distributions 2. Control nitrogen- and sulfur-containing products, tar, and gaseous emissions in fuel-oriented routes, while improving upgrading efficiency and catalyst stability 3. Increase hydrothermal product selectivity, limit secondary degradation of amino acids and sugars, and develop separation and use of aqueous products 4. Establish continuous operation, mass and energy balances, and techno-economic and environmental assessments
Functional carbon materials	1. De-oiled rapeseed meal 2. Solid residues remaining after rapeseed meal fractionation	1. Adsorption performance of activated carbons 2. Electrocatalytic evaluation of nitrogen- and sulfur-doped carbon materials 3. Solid-acid catalysis and reuse	1. Relate feedstock composition and pyrolysis and activation conditions to pore structure, nitrogen and sulfur speciation, and target performance 2. Increase the yield of usable carbon materials while reducing activation-agent consumption, washing-water demand, and wastewater generation 3. Validate regeneration, cycling stability, and service life of adsorbents and catalysts

## Data Availability

No new data were created or analyzed in this study. Data sharing is not applicable to this article.
